# Loss of KDM5A-mediated H3K4me3 demethylation promotes aberrant neural development by Wnt/β-catenin pathway activation

**DOI:** 10.1038/s41419-025-08208-5

**Published:** 2025-11-20

**Authors:** Jianting Li, Yuxiang Liang, Zhihua Cao, Qi Xie, Xiaolong Gu, Peng Ma, Lu Li, Wenbin Ren, Yuan Fu, Zhizhen Liu, Yonghong Wang, Ting Zhang, Hailan Yang, Shuyan Yang, Jun Xie

**Affiliations:** 1 Department of Biochemistry and Molecular Biology, College of Basic Medicine, Shanxi Key Laboratory of Birth Defect and Cell Regeneration, MOE Key Laboratory of Coal Environmental Pathogenicity and Prevention, Shanxi Medical University, Taiyuan, China; 2 Shanxi Key Laboratory of Human Disease and Animal Models, Experimental Animal Center of Shanxi Medical University, Taiyuan, Shanxi China; 3 Department of Gynaecology and Obstetrics, The Second Hospital of Shanxi Medical University, Taiyuan, China; 4 Beijing Municipal Key Laboratory of Child Development and Nutriomics, Capital Center for Children’s Health, Capital Institute of Pediatrics, Capital Medical University, Beijing, China; 5 Department of Obstetrics, First Clinical College of Shanxi Medical University, Taiyuan, China

**Keywords:** Neural tube defects, Developmental neurogenesis

## Abstract

Neural tube defects (NTDs) are common and severe birth defects. Folate supplementation can prevent NTDs, but the underlying molecular mechanisms are unclear. Aberrant wnt/β-catenin pathway activation leads to defective anteroposterior patterning, resulting in NTDs, but little is known about whether epigenetic factors contribute to this process. Here, we performed ChIP and Cut&Tag to explore H3K4me3 in folate-deficient cells and NTDs mouse models. Our findings show folate deficiency increased H3K4me3 levels at wnt target genes promoters, enhancing their transcription. This effect was mediated by reduced expression of histone demethylase KDM5A, leading to the maintenance of H3K4me3 marks and activation of wnt/β-catenin signalling. Similarly, wnt/β-catenin pathway was activated in KDM5A-KO cells, differentiation of neuronal progenitors cells from mouse ESCs under folate deficiency and folate-deficient NTD mice. Intriguingly, KDM5A depletion in zebrafish embryos resulted in defective neurodevelopment and increased wnt signalling. Furthermore, the transcription factor PAX2 downregulated KDM5A under folate-deficient conditions. Clinically, increased H3K4me3 levels and wnt target genes expression were observed in low-folate NTDs brain samples. All these findings suggest KDM5A-dependent epigenetic regulation of wnt signaling is crucial in low folate NTDs, implicating a potential therapeutic target.

## Introduction

Neural tube defects are severe birth defects that are thought to be associated with genetic and environmental factors. They result from failure of neural tube closure during the first trimester [1]. Supplementation with folate before pregnancy can prevent 30–70% of NTDs [2, 3]. Although our understanding of NTDs has recently improved greatly, the aetiology and molecular mechanisms underlying the preventive effect of folate remain unclear, and this lack of clarity impedes the development of improved therapeutic interventions.

During neural development, tightly regulated signalling pathways are critical for morphogenesis. The non-canonical Wnt/PCP pathway is essential for neural tube closure via regulating convergent extension. In addition, the canonical Wnt/β-catenin signalling governs anteroposterior patterning by modulating neural progenitor proliferation and differentiation. Dysregulation of either pathway may contribute to NTDs through distinct mechanisms [4, 5]. Numerous reports have highlighted the importance of Wnt/β-catenin signalling in embryonic neurodevelopment [6–8]. For example, Wnt1 and Wnt3a are expressed at the dorsal midline of the developing neural tube [9]. In addition, aberrant activity of the canonical Wnt/β-catenin pathway leads to defective anteroposterior patterning [10]. Our previous work identified hyperactivation of Wnt/β-catenin signalling in a folate-deficient mouse model, mediated by the Gcm1/β-catenin/TCF4 complex [11], yet the upstream triggers for this dysregulation in NTDs are not fully defined.

Epigenetic mechanism, particularly posttranslational modifications, have emerged as key regulators of gene expression programs during development [12, 13]. Among them, trimethylation of histone H3 at lysine 4 (H3K4me3) is a prominent activation mark enriched at the transcription start sites to facilitate RNA polymerase II (RNAPII) binding and gene activation [14, 15]. Critically, alterations in sperm H3K4me3 can be paternally inherited and linked to aberrant gene expression and increased severity of birth defects in offspring, highlighting its developmental significance [16, 17]. A connection between H3K4me3 and Wnt signalling has been suggested in diseases [18, 19]. For example, *Bam Marpe* et al. revealed that the Wnt signalling pathway is upregulated in immune cells of posttraumatic stress disorder patients as a result of epigenetic changes involving the histone modification H3K4me3, which in turn may promote an inflammatory phenotype in these cells [20]. However, it is currently unknown whether H3K4me3 regulates the Wnt/β-catenin signalling pathway and, if so, whether this regulation is important for neural tube development.

The dynamic control of H3K4me3 is mediated by methyltransferases and demethylases. The H3K4 demethylase KDM5A (also known as JARID1A/RBP2) functions as a transcriptional repressor by removing di- and trimethyl groups [21]. Accumulating evidence suggests that KDM5A plays essential roles in mitochondrial metabolism, cell cycle progression, cellular senescence, development and differentiation, and tumorigenesis [22–25]. However, the role of KDM5A in early development is poorly understood. It reported the active removal of methylation from broad H3K4me3 domains by the lysine demethylases KDM5A and KDM5B is required for normal zygotic genome activation and early embryonic development [26]. Notably, PI3K/AKT signalling has been shown to regulate H3K4 methylation and the transcription of cell cycle-promoting genes via a KDM5A-dependent mechanism [27]. Most intriguingly, KDM5A can inhibit the expression of Wnt6 by decreasing the level of the activating epigenetic marker H3K4me3 in the Wnt6 promoter, which leads to inhibition of the Wnt/β-catenin pathway and activation of the adipogenic program [28]. Despite these findings, the function of KDM5A in neural development and its potential involvement in NTD pathogenesis, particularly through the modulation of Wnt/β-catenin signalling, is completely unexplored.

Therefore, we hypothesized that KDM5A, by demethylating H3K4me3 at critical gene promoters, serves as a key repressor of the Wnt/β-catenin pathway during neural tube development, and its loss leads to pathway hyperactivation and NTDs. The aim of the present study was to identify novel regulatory targets or strategies for the diagnosis and early prevention of NTDs. By using an integrated approach including Cut&Tag and luciferase reporter assays, we elucidated the mechanism by which loss of KDM5A-mediated H3K4me3 demethylation promotes aberrant neural development through wnt/β-catenin pathway activation. Our findings not only improve the aetiological understanding of folate-sensitive NTDs but also provide new insights and potential targets for related congenital birth defects.

## Results

### H3K4 methylation correlate with the upregulation of wnt/β-catenin target genes under folate deficiency

**Fig. 1:**
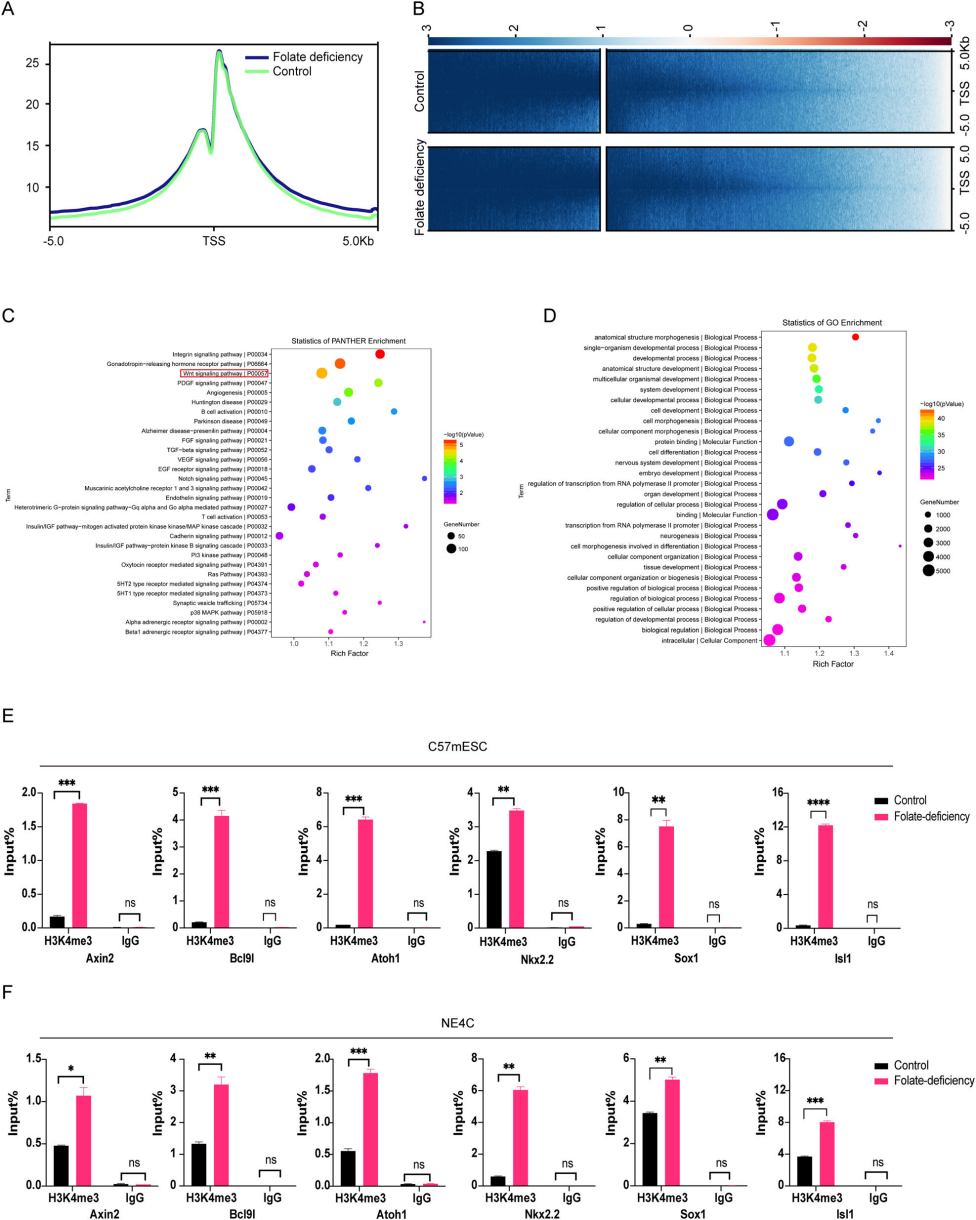
Increased H3K4me3 levels induced by folate deficiency bind neurodevelopment-related gene promoters in the wnt/β-catenin pathway. **A** Comparison of average H3K4me3 ChIP-seq read densities between control and folate-deficient C57mESCs. **B** H3K4me3 ChIP-seq enrichment profiles of control and folate-deficient mESCs. **C** Pathway analysis of H3K4me3 ChIP peaks via PANTHER. The x-axis shows the enrichment factor, the y-axis shows the binomial raw *P* value, and the size of each circle represents the number of genes in the pathway. **D** GO enrichment analysis of H3K4me3 ChIP peaks. The x-axis shows the enrichment factor, the y-axis shows the binomial raw *P* value, and the size of each circle represents the number of genes in the GO term. **E** H3K4me3 ChIP assays were performed using C57mESCs subjected to folate deficiency for 6 passages. Mouse IgG was used as a control. The enrichment of H3K4me3 in the promoters of the neurodevelopment-related genes *Axin2, Bcl9l, Atoh1, Nkx2.2, Sox1* and *Isl1* was measured via RT‒qPCR. *p < 0.05, **p < 0.01, ***p < 0.001, ****p < 0.0001. **F** H3K4me3 ChIP assays were performed using mouse NE4C cells subjected to folate deficiency for 6 passages. Mouse IgG was used as a control. The enrichment of H3K4me3 in the promoters of the neurodevelopment-related genes *Axin2, Bcl9l, Atoh1, Nkx2.2, Sox1* and *Isl1* was measured via RT‒qPCR. *p < 0.05, **p < 0.01, ***p < 0.001. **E**, **F** are presented as the means ± SEMs from three biological replicates.

Mouse postnatal paternal folate deficiency alters H3K4me3 at developmental loci in sperm, and folate deficiency in KDM1A transgenics mouse increases sperm H3K4me3 levels and the likelihood of birth defects [16]. Our previous studies also demonstrated that folate deficiency activates wnt/β-catenin signalling in mESCs [11]. To screen for developmental genes involved in wnt/β-catenin signalling that are regulated by H3K4me3, we performed H3K4me3 ChIP-seq in mESCs cultured under folate-deficient conditions. Overall, at the global genome level, the signals did not differ significantly between the folate-deficient and control conditions (Fig. 1A, B). Thus, we screened specifically differential methylated peaks between the two conditions for further enrichment analysis. Pathway enrichment for differential methylated peaks analysis revealed the wnt/β-catenin signalling pathway was one of the three most enriched pathways among the top 30 pathways, which is consistent with our published folate-deficient C57mESC agilent mouse mRNA array data [11] (Fig. 1C). Additionally, Gene Ontology (GO) enrichment for differential methylated peaks analysis revealed that the genes with H3K4me3 modifications were overrepresented among genes related to embryo development and neurogenesis (Fig.1D). These results suggested that H3K4me3 might play a pivotal role in the wnt/β-catenin signalling pathway to mediate development.

We demonstrated in our previous study that the wnt/β-catenin signalling pathway is activated under conditions of folate deficiency. To explore the role of H3K4me3 in this process, target genes related to neurodevelopment and involved in wnt/β-catenin signalling were analysed. ChIP‒qPCR revealed increased H3K4me3 methylation around (within 2 kb) the promoter regions of *Axin2, Bcl9l, Atoh1, Nkx2.2, Sox1* and *Isl1* in folate-deficient C57mESCs and neuro epithelial clone 4 C cell (NE4C), which are better simulate the biological functions of neural tube development (Fig. 1E, F). We next performed RT‒qPCR analysis of all the abovementioned neurodevelopment-related genes and found that all these genes were upregulated in folate-deficient NE4C cells (Supplementary Fig. 1A). Furthermore, after MTX, a folate antagonist, was used to cause folate deficiency, RT‒qPCR revealed that all these neurodevelopment-related genes were upregulated and reversed by folate supplementation, but except for Tcf12 (Supplementary Fig. 1B). Taken together, these data suggest that the increased expression of these neurodevelopment-related genes in the wnt/β-catenin signalling pathway may be attributed to the increases in their H3K4me3 signals under folate-deficient conditions, as the presence of H3K4me3 is associated with the upregulation of nearby genes because chromatin accessibility is facilitated by histone methylation [29].

### Decreased KDM5A expression is responsible for H3K4me3 modification of wnt/β-catenin target genes in the context of folate deficiency

**Fig. 2:**
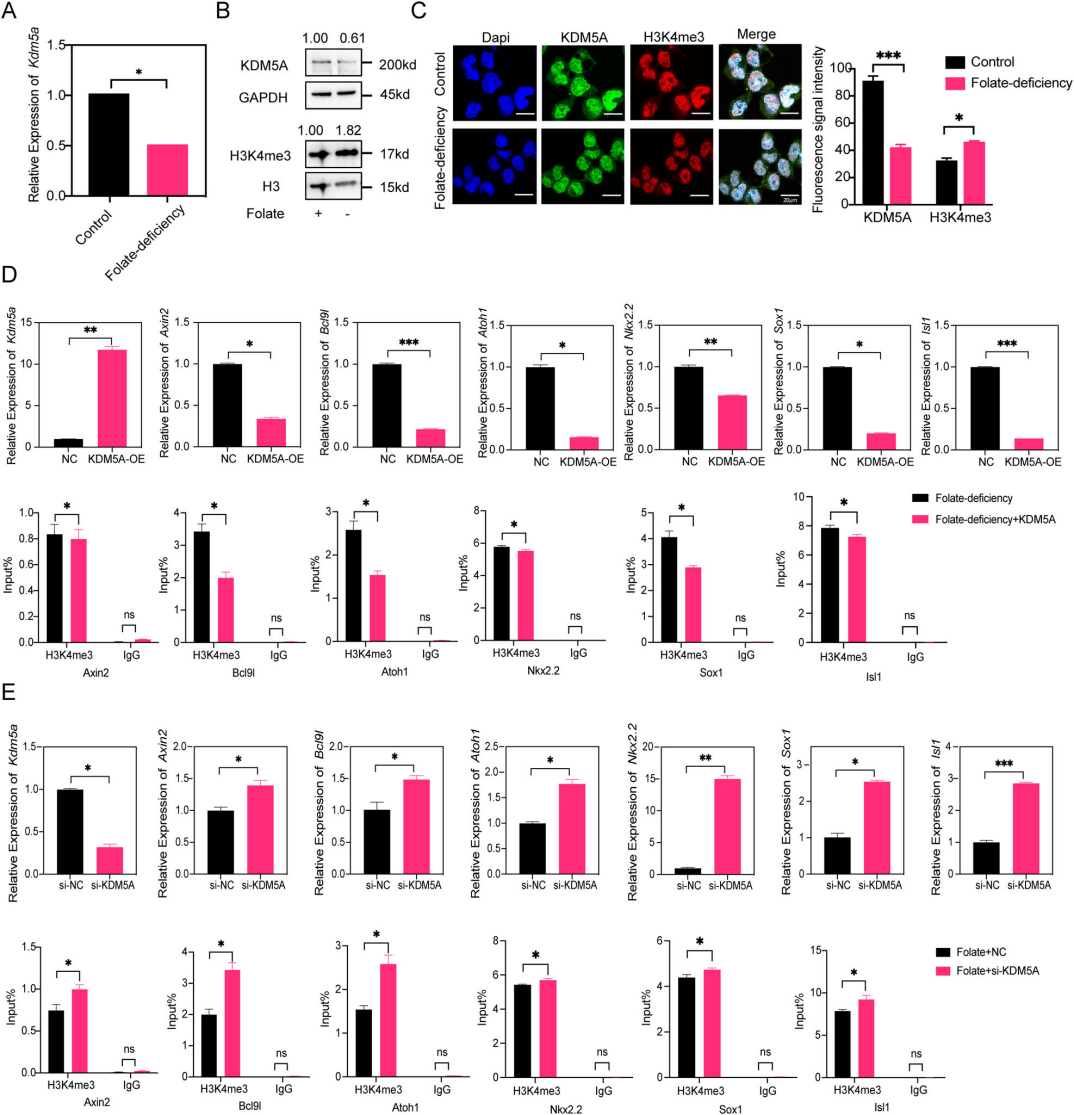
KDM5A is required for the upregulation of neurodevelopment-related genes in the wnt/β-catenin pathway in the setting of folate deficiency. **A** *Kdm5a* mRNA expression in folate-deficient NE4C cells. The value was normalized to the mRNA level of *Gapdh* in the same sample. *p < 0.05. **B** KDM5A and H3K4me3 levels in folate-deficient NE4C cells were measured via Western blotting. GAPDH and H3 were used as loading controls for KDM5A and H3K4me3, respectively. **C** Immunostaining for KDM5A and H3K4me3 in folate-deficient NE4C cells. Images were acquired with a confocal microscope, and the nuclei were stained with DAPI. *p < 0.05, ***p < 0.001. **D** RT‒qPCR analysis of neurodevelopment-related gene expression in NE4C cells with folate deficiency and overexpression of KDM5A. NE4C cells subjected to folate deficiency were transfected with the KDM5A overexpression plasmid for 48 h and then collected for analysis. *Gapdh* was used as a loading control (upper panel). H3K4me3 ChIP assays were performed in NE4C cells with folate deficiency and KDM5A overexpression. Mouse IgG was used as a loading control (lower panel). *p < 0.05, **p < 0.01, ***p < 0.001. **E** RT‒qPCR analysis of neurodevelopment-related gene expression in NE4C cells after knockdown of KDM5A. NE4C cells were transfected with si-KDM5A for 48 h and then collected for analysis. *Gapdh* was used as a loading control (upper panel). H3K4me3 ChIP assays were performed in NE4C cells after siKDM5A transfection. Mouse IgG was used as a loading control (lower panel). *p < 0.05, **p < 0.01, ***p < 0.001. All the above data are presented as the means ± SEMs from three biological re*p*licates.

We further investigated the reason that the H3K4me3 levels in wnt/β-catenin target genes are increased upon folate deficiency. A possible reason is that the expression of some proteins associated with lysine demethylation is decreased upon folate deficiency. To test our hypothesis, we searched for the most common candidate H3K4me3 demethylases, including KDM5A and KDM5B. WB analysis was performed to evaluate the protein expression of KDM5A and KDM5B in folate-deficient C57mESCs and HEK293T cells separately. The protein level of KDM5A but not that of KDM5B was significantly decreased under folate-deficient conditions (Supplementary Fig. 2A and Fig. 2B). These findings suggest that H3K4me3 demethylation is regulated by KDM5A but not KDM5B. To confirm this result, we also measured KDM5A and H3K4me3 levels in folate-deficient NE4C cells via WB analysis. The results revealed an increased H3K4me3 levels and decreased KDM5A protein levels (Fig. 2A).

Next, we evaluated the KDM5A and H3K4me3 levels upon folate deficiency in a folate-deficient NE4C cell model as previously reported [11]. First, we found that the *Kdm5a* mRNA level measured via RT‒qPCR was lower in the folate-deficient group than in the untreated group (Fig. 2A). We also performed WB analysis to validate the KDM5A and H3K4me3 protein levels. Consistent with the above findings, the KDM5A protein level decreased while the H3K4me3 level increased in the folate-deficient group (Fig. 2B). Additionally, the results of immunofluorescence staining were similar to those of the WB analysis, revealing a decrease in KDM5A expression and a notable increase in the H3K4me3 levels (Fig. 2C). To further validate the removal of H3K4me3 by KDM5A in the presence and absence of folate, KDM5A siRNAs and a KDM5A overexpression plasmid were used separately to modulate the KDM5A level in NE4C cells. Notably, RT‒qPCR analysis revealed that Wnt target gene mRNA expression was significantly decreased in NE4C cells overexpressing KDM5A mRNA overexpression cultured under folate-deficient conditions (Fig. 2D, upper panel). Next, we examined the level of H3K4me3 in the promoters of the selected Wnt target genes via ChIP‒qPCR. As shown in Fig. 2D, the H3K4me3 levels were lower in the promoter regions of the selected target genes than in those of the untreated group. These findings suggest that the demethylase activity of KDM5A is regulated by folate. To confirm this result, we further measured *Kdm5a* and Wnt target gene mRNA levels in NE4C cells cultured under normal folate conditions and modified them with siRNAs. The transcription of selected Wnt target genes was activated after KDM5A was significantly knocked down (Fig. 2E, upper panel). Moreover, the H3K4me3 levels in the promoter regions of the selected target genes were increased (Fig. 2E, lower panel).

To further confirm our results, we also established a folate-deficient 293 T cell model via the same methods used to establish the NE4C cell model. After sixth passage, no significant differences in the total number of cells or cell morphology were observed between the two groups (Supplementary Fig. 2C). The intracellular folate levels in folate-deficient HEK293T cells were significantly lower than those in control cells (Supplementary Fig. 2D). RT‒qPCR analysis revealed that the *Kdm5a* mRNA level was lower in the folate-deficient group than in the untreated group (Supplementary Fig. 2E). We subsequently measured the KDM5A protein and H3K4me3 levels via WB analysis. The KDM5A protein level decreased, but the H3K4me3 level was increased under folate-deficient conditions (Supplementary Fig. 2F). We further revealed that KDM5A overexpression led to the removal of H3K4me3, whereas the H3K4me3 level was increased after KDM5A siRNA transfection (Supplementary Fig. 2G). These changes were consistent with the changes in the transcription of wnt target genes after KDM5A overexpression and knockdown upon folate deprivation in NE4C cells. Overall, our findings indicate that decreased KDM5A expression is required for wnt/β-catenin target gene expression through increases in H3K4me3 levels at the genomic loci of these selected promoters under folate-deficient conditions.

### KDM5A deficiency and folate deprivation disrupt H3K4me3 landscapes on neurodevelopmental regulators

**Fig. 3:**
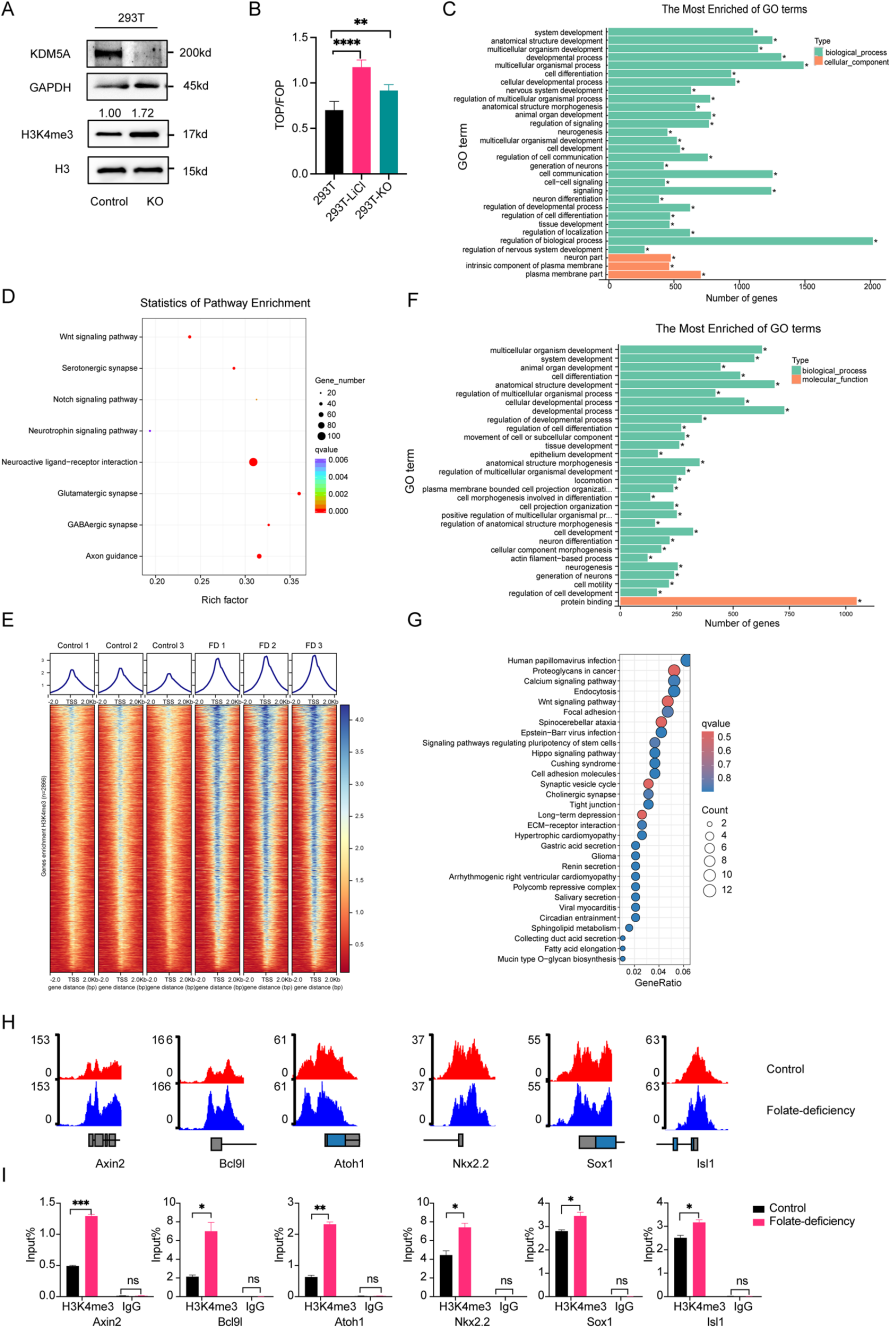
H3K4me3 is enriched in wnt signalling pathway genes and neurodevelopment-related genes upon KDM5A knockout and folate-deficient NPSCs. **A** KDM5A and H3K4me3 levels in KDM5A-KO 293 T cells were measured via Western blotting. GAPDH and H3 were used as loading controls for KDM5A and H3K4me3, respectively. **B** Analysis of luciferase activity via TOP Flash/FOP Flash assays in KDM5A-KO 293 T cells. 293 T cells treated with 10 mM LiCl for 24 h were used as a positive control. ***p < 0.01, ***p < 0.001. **C** GO enrichment analysis of genes with H3K4me3 peaks identified by Cut&Tag. The x-axis shows the number of genes, and the y-axis shows the GO terms. **D** KEGG pathway enrichment analysis of genes with H3K4me3 peaks identified by Cut&Tag. The x-axis shows the Rich factor, the y-axis shows the binomial pathway, and the size of each circle represents the number of genes. **E** Differential H3K4me3 peaks at TSS between control and NPSCs under folate deficiency. FD: folate deficiency. **F** GO enrichment analysis of genes with H3K4me3 peaks in NPSCs. The x-axis shows the number of genes, and the y-axis shows the GO terms. **G** KEGG analysis of genes with H3K4me3 peaks in NPSCs. The x-axis shows the Gene Ratio, the y-axis shows the binomial pathway, and the size of each circle represents the number of genes. **H** IGV visualization showing the differential H3K4me3 peaks of Wnt target genes promoters in NPSCs with folate deficiency (2 kb upstream and downstream of the TSS). **I** H3K4me3 ChIP assays were performed on NPSCs with folate deficiency. Mouse IgG was used as a control. The enrichment of H3K4me3 in the promoters of the Wnt target genes *Axin2, Bcl9l, Atoh1, Nkx2.2, Sox1* and *Isl1* was measured via RT‒qPCR. *p < 0.05, **p < 0.01, ***p < 0.001. Figure 3A, 3B and 3I are presented as the means ± SEMs from three biological replicates.

To further clarify how the regulation of H3K4 methylation by the histone demethylase KDM5A contributes to wnt/β-catenin target gene expression, we constructed a KDM5A-KO 293 T cell line via CRISPR/Cas9 genome editing (Supplementary Fig. 3A). WB analysis revealed that KDM5A was indeed strongly knocked down and that the H3K4me3 level was increased (Fig. 3A). To verify the change in wnt activity in KDM5A-KO 293 T cells, we performed a TOP Flash/FOP Flash luciferase assay. The transcriptional activity of the TOP Flash/FOP Flash reporters was significantly increased in KDM5A-KO 293 T cells stably overexpressing the TOP Flash reporter or FOP Flash reporter (Fig. 3B). Similar results were observed in folate-deficient C57mESCs and folate-deficient NE4C [11]. We also investigated the genome-wide distribution of H3K4me3 binding sites in KDM5A-KO cells via Cut&Tag. GO enrichment analysis revealed that the genes with enriched with H3K4me3 tended to be involved in nervous system development (Fig.3C). Furthermore, Kyoto Encyclopaedia of Genes and Genomes (KEGG) pathway enrichment analysis revealed that including the wnt signalling pathway, axon guidance, glutamatergic synapse, and other nervous signalling pathway could be enriched (Fig.3D). These results indicate that KDM5A knockout activates the wnt pathway, which likely influences aberrant neurodevelopment.

To elucidate H3K4me3 epigenetic role in neurodevelopment, we also established a folate-deficient mESC differentiating into neuronal progenitors (Supplementary Fig. 3B). Then, we conducted H3K4me3 Cut&tag on NPSCs to analysis the epigenetic role influenced by folate. Differential peak enrichment analysis of H3K4me3 at transcription start sites (TSS) showed a global gain in H3K4me3 with folate deficiency (Fig. 3E). KEGG pathway analysis of the differential H3K4me3 peaks revealed significant enrichment in the wnt and Hippo signaling pathways (Fig.3F). GO analysis also revealed that differenced H3K4me3 driven by folate deficiency mainly enriched in system development, organ development, neurogenesis and neuron differentiation and so on (Fig.3G). Consistent with these findings, elevated H3K4me3 modification on wnt target genes was stronger in NPSCs with folate deficiency compared to control group (Fig.3H). ChIP-qPCR also confirmed the same H3K4me3 enrichment in wnt target genes of NPSCs with folate deficiency (Fig.3I). These results suggest folate deficiency disrupts the epigenetic landscape of H3K4me3 in neural progenitor cells, leading to aberrant hyper-methylation on wnt signaling and potentially impairing neuronal differentiation. In summary, KDM5A loss and folate deficiency cooperatively disrupt H3K4me3, compromising the epigenetic regulation of neurodevelopmental process.

### A low level of the demethylase KDM5A results in aberrant neurodevelopment in NTD animal models

**Fig. 4:**
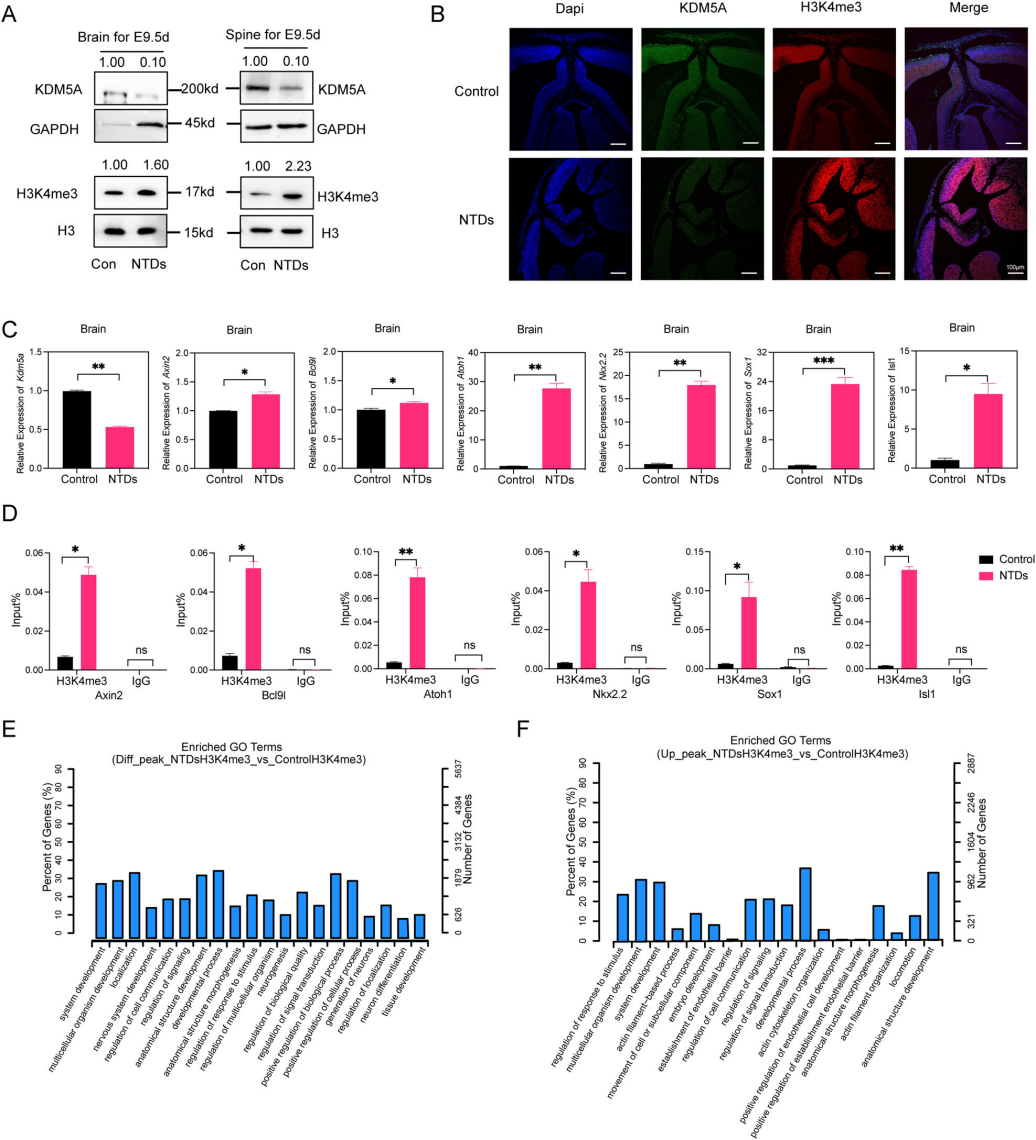
Folate deficiency increases H3K4me3 levels in NTD mice. **A** Morphology of CD-1 mouse embryos with low-folate diet and MTX induced from E9.5, representative images were shown, left panel is control mouse fetus(n = 100), middle panel is anencephalous(n = 26), and right panel is spina bifida (n = 18). The arrowhead is where the NTDs located. Scale bar, 1 mm. **B** Immunostaining for KDM5A and H3K4me3 in the neural tubes in E13.5 folate-deficient NTD mouse embryos(n = 6) and control embryos(n = 6). Images were acquired with a confocal microscope, and nuclei were stained with DAPI. **C** RT‒qPCR analysis of neurodevelopment-related gene expression in the brains of folate-deficient NTD model mice at E9.5(n = 6) and control mice(n = 6). *Gapdh* was used as a loading control. *p < 0.05, **p < 0.01, ***p < 0.001. **D** H3K4me3 ChIP assays were performed in brain tissue from E9.5 folate-deficient NTD model mice(n = 12) and control mice(n = 12). Mouse IgG was used as a control. The enrichment of H3K4me3 in the promoters of the neurodevelopment-related genes *Axin2*, *Bcl9l*, *Atoh1*, *Nkx2.2*, *Sox1* and *Isl1* was measured via RT‒qPCR. *p < 0.05, **p < 0.01, ***p < 0.001. **E** KDM5A and H3K4me3 levels in the folate-deficient NTD mouse model(n = 12) and control mice(n = 12) brains and spines at E9.5 were measured via western blotting. GAPDH and H3 were used as loading controls for KDM5A and H3K4me3, respectively. **F** GO enrichment analysis of genes with increased H3K4me3 peaks identified by Cut&Tag in E9.5 folate-deficient NTD model mice. The x-axis shows the GO terms, and the Y-axis shows the percentage of genes. Fig. 4C and 4D are presented as the means ± SEM from three biological replicates.

To investigate how KDM5A comprehensively regulates aberrant neurodevelopment, we established a mouse model of low-folate-induced NTDs that was described previously [11]. Considering that nervous system development in mice is complete by day 10.5 of embryonic development (E10.5), we examined neural tissue at E9.5. Compared with control mice, NTD model mice presented severe spina bifida and anencephaly (Fig. 4A). Additionally, immunohistochemical analysis of brain tissue from E13.5 embryos from NTD model mice revealed similar findings (Supplementary Fig. 4A). Next, immunofluorescence staining revealed that in neural tube tissues from E9.5 embryos in the NTD model, the KDM5A level was decreased and the H3K4me3 level was significantly increased (Fig. 4B). Additionally, to further confirm the role of the wnt signalling pathway in NTDs, we performed RT‒qPCR analysis of wnt target genes in both the brains and spines of E9.5 embryos in the NTD model and found that all six tested wnt target genes were upregulated, but that *Kdm5a* was downregulated in the brains of these embryos (Fig. 4C). The same results were observed in the spines of embryos in the NTD mice model (Supplementary Fig. 4C). We then performed ChIP‒qPCR with an anti-H3K4me3 antibody in the brain tissue of E9.5 embryos in the NTD model and detected significant H3K4me3 enrichment in the promoters of wnt target genes compared with that in the IgG control group (Fig. 4D). Finally, to evaluate protein levels in the brains and spines of NTD model mice, we performed WB analysis of KDM5A and H3K4me3. Compared with that in control mice, the protein level of KDM5A was significantly decreased but the H3K4me3 level was increased in the brains and spines of NTD model mice (Fig. 4E). All these results suggested down-regulated KDM5A demethylated H3K4me3 in E9.5 mice model.

To confirm above result, we also performed H3K4me3 Cut&Tag with E9.5 d mice brain. H3K4me3-Tags from E9.5 d NTDs mouse brain also revealed that differenced H3K4me3 peaks were enriched in system development and nervous system development (Supplementary Fig. 4B). Specifically, we mainly focus on the up H3K4me3 peaks in E9.5 d NTDs mouse brain, GO enrichment analysis revealed that both them were mostly in genes related to system development and embryo development (Fig. 4F). Considering the results in Fig.4D, these results preliminarily again indicated that a low KDM5A level resulted in the activation of wnt target gene expression through increases in the H3K4me3 levels in the corresponding promoters in E9.5 d NTD mice model.

### KDM5A knockdown impaired brain neurodevelopment in embryos

**Fig. 5:**
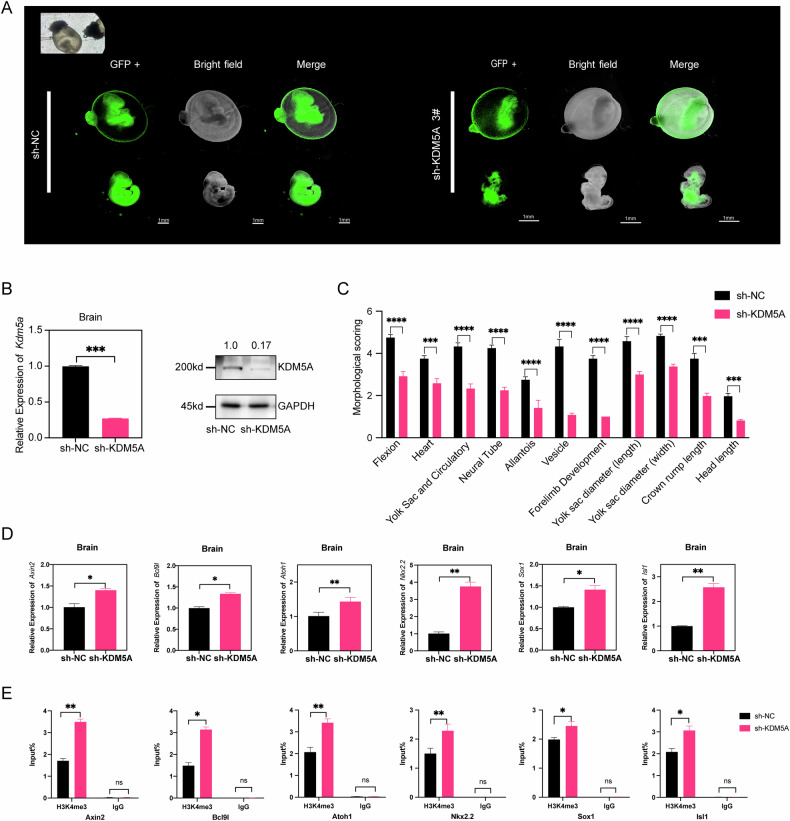
KDM5A knockdown with lentiviral microinjection impairs brain neurodevelopment in mouse embryos. **A** Morphology of E10.5 CD-1 mouse embryos were injected with control sh-NC lentivirus (n = 30) or sh-KDM5A lentivirus(n = 26), respectively. Freshly dissected E8.5 embryos were microinjected with sh-KDM5A GFP lentivirus with target 3 (titer~1 × 10⁹ TU/mL) or control sh-NC GFP lentivirus (titer ~1 × 10⁹ TU/mL). Then these embryos were cultured in WEC for 48 h, reaching the equivalent of E10.5 for embryo analysis. Scale bar, 1 mm. The bright field image of microinjection was shown in the upper left corner. **B** RT‒qPCR analysis (left panel) and western blotting (right panel) of E10.5 d mouse brains with sh-KDM5A lentivirus (n = 8) knockdown and sh-NC lentivirus(n = 8). *Gapdh* was used as loading controls. ***p < 0.001. **C** Morphological scoring of E10.5 d mouse embryos comparison between control sh-NC lentivirus (n = 30) and sh-KDM5A lentivirus knockdown (n = 26). Bar graph showing morphological scoring of various developmental structures and measurements in embryos with control and KDM5A lentivirus knockdown. Features evaluated include flexion, heart development, yolk sac and circulatory system, neural tube formation, allantois, vesicle, forelimb development, yolk sac diameter (length and width), crown-rump length, and head length. *p < 0.05, **p < 0.01, ***p < 0.001, ****p < 0.0001. **D** RT‒qPCR analysis of neurodevelopment-related gene expression in the brains of mice at E10.5 with control sh-NC (n = 4) lentivirus or sh-KDM5A(n = 4) lentivirus. GAPDH was used as a loading control. *p < 0.05, **p < 0.01, ***p < 0.001. **E** H3K4me3 ChIP assays were performed in brain tissue from E10.5 mice with KDM5A knockdown with control sh-NC lentivirus (n = 18) or sh-KDM5A lentivirus (n = 14). Mouse IgG was used as a control. The enrichment of H3K4me3 in the promoters of the neurodevelopment-related genes *Axin2, Bcl9l, Atoh1, Nkx2.2, Sox1* and *Isl1* was measured via RT‒qPCR. *p < 0.05, **p < 0.01, ***p < 0.001. All the above data are presented as the means ± SEM from three biological replicates.

To further validate the role of low KDM5A levels in embryos neurodevelopment, we constructed sh-KDM5A lentivirus and chosen effective lentivirus to perform microinjection in E8.5 d mouse embryos, which is important for neural tube development in this stage (Supplementary Fig. 5A and B). Then we used three-dimensional roller culture system-whole embryo culture (WEC) to extended culture these embryos for another 48 hours, reaching the equivalent of E10.5 d where the neural tube closure was finished. The results showed compared with empty lentivirus vector microinjection, the mouse embryos with sh-KDM5A lentivirus microinjection in E10.5 d exhibited severe development delays and defects including abnormal brain development and aberrant open spinal curling (Fig. 5A). To elucidate KDM5A role in brain development, RT-PCR and WB revealed again that KDM5A was knockdown efficiently in the aberrant brains in E10.5 d (Fig.5B). Morphological scoring across different developmental systems, including the neural tube, revealed a significantly decreased score in the sh-KDM5A groups (Fig. 5C), indicating that KDM5A plays a vital role in neural tube closure. RT-qPCR also revealed all six wnt targets expression gene were upregulated (Fig. 5D). At last, ChIP-qPCR of E10.5 d embryos after KDM5A knockdown also showed increased H3K4me3 peaks in six wnt targets genes promoters (Fig. 5E). All these results suggested KDM5A knockdown is related to active wnt genes expressions and aberrant neural development.

**Fig. 6:**
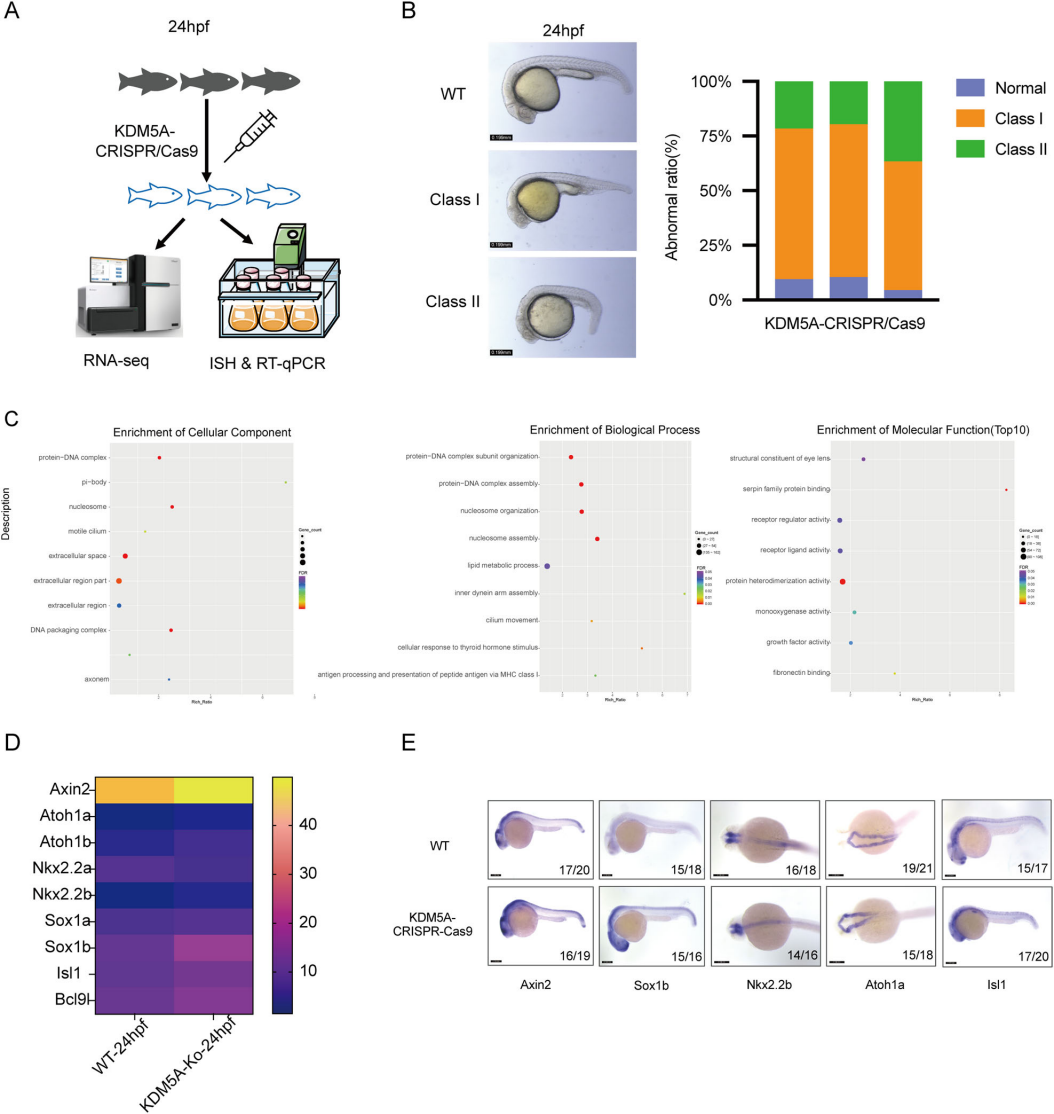
Wnt targets genes were upregulated in KDM5A-KO zebrafish. **A** Scheme of experiment design upon 24hpf zebrafish with KDM5A-CRISPR/Cas9. **B** Morphology of 24hpf zebrafish embryos with KDM5A knocking down with CRISPR/Cas9. Scale bar, 0.19 mm. The class I and Class II showed NTDs-like aberrant neurodevelopment. The abnormal ratio of 24hpf zebrafish embryos with KDM5A knocking down were showed in right panel with three replicate experiments. **C** GO enrichment analysis in biological process, cellular component and molecular function of different genes upon KDM5A knocking down with CRISPR/Cas9. The x-axis shows the Rich Ratio the y-axis shows the binomial pathway, and the size of each circle represents the number of genes. **D** Heatmap analysis of wnt target genes in 24hpf zebrafish upon KDM5A knockout with CRISPR/cas9. **E** In situ hybridization for Wnt target genes in the 24hpf zebrafish with KDM5A CRISPR/Cas9 injection. The size of zebrafish used for experiment were shown in the lower right corner of each image. The denominator represents the total number of samples, and the numerator represents the number of samples with positive results respectively.

Next, we also used CRISPR/Cas9 injection to knock down *Kdm5a* in zebrafish embryos, which offers many experimental advantages for in vivo imaging and genetic studies [30]. At 24 hpf, the morphants injected with KDM5A-CRISPR/Cas9 presented phenotypic traits similar to KDM5A knockdown and those observed in the folate-deficient NTD mouse model, including a curved body axis and a reduced head size (Figs. 5A and 6B). To screen different genes in 24hpf zebrafish with KDM5A-knocking down, we performed RNA-seq for 24hpf zebrafish with KDM5A-knocking down with neural tube finishing at this stage. There are 1959 up-regulated genes, and 729 down-regulated genes were screened out (Supplementary Fig. 6A). GO enrichment analysis showed that different genes can be enriched in synapse part, developmental process, growth, transcription regulator activity(Fig. 6C). We further evaluated the mRNA expression of Wnt target genes in WT and KDM5A-knocking down zebrafish. The result showed most of these genes were upregulated with KDM5A-knocking down, consistent with RT-qPCR results (Fig. 6D and Supplementary Fig. 6B). At last, we also further choose several genes mRNA levels by ISH, the results also showed a significantly upregulation of *Axin2*, *Sox1a*, *Nkx2.2b*, *Atoh1a* and *Isl1*. All these findings in our KDM5A-KO zebrafish model were consistent with those in NTD mice model, indicating that KDM5A knockdown disrupts processes involved in neurodevelopment again. Collectively, these results confirmed that KDM5A is the mutated gene responsible for the aberrant neurodevelopment phenotype in zebrafish.

### PAX2 regulates KDM5A transcription in the setting of folate deficiency

**Fig. 7:**
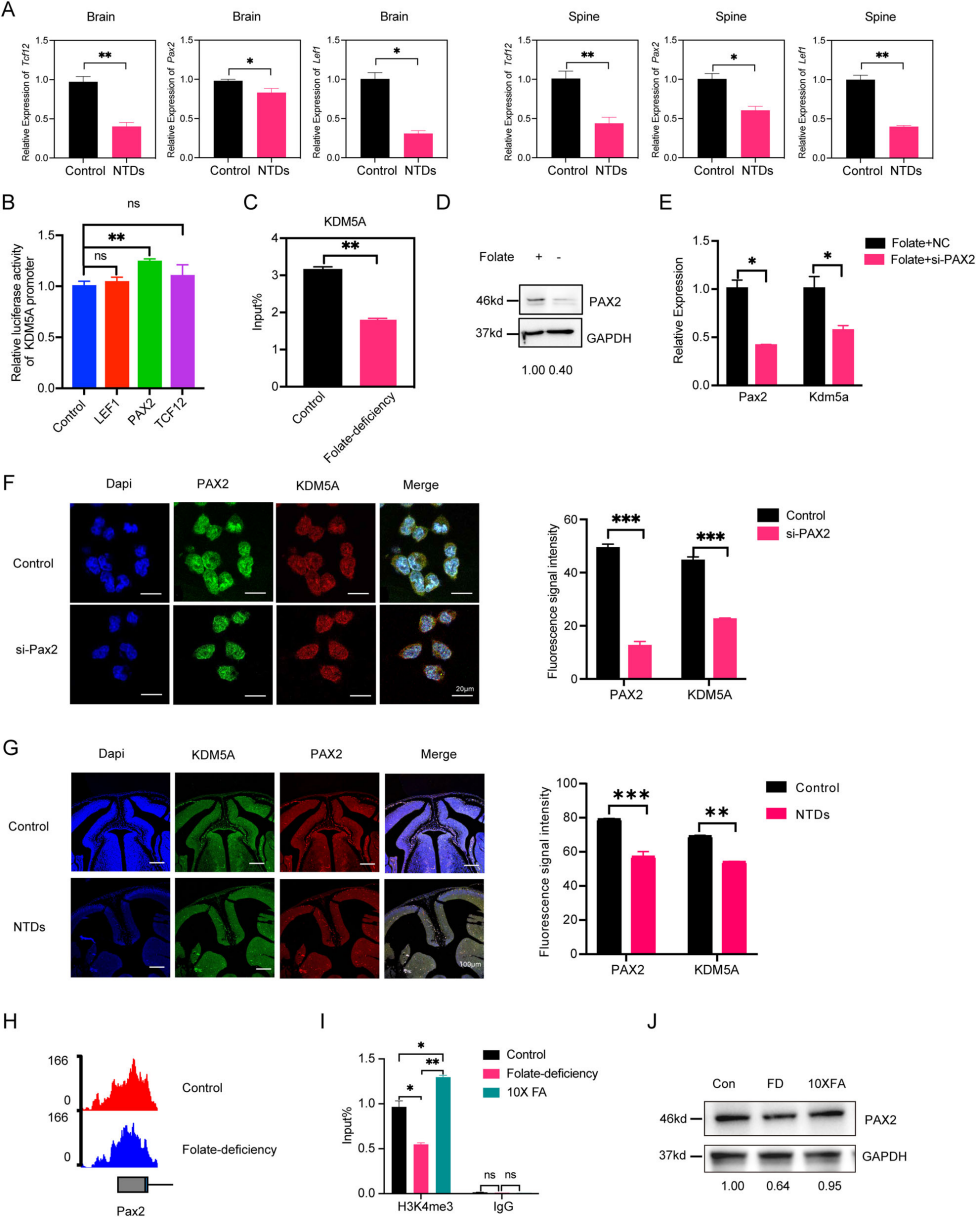
PAX2 regulates Kdm5a promoter activity in the context of folate deficiency. **A** RT‒qPCR analysis of *Tcf12*, *Pax2*, and *Lef1* mRNA expression in the spines and brains of E9.5 folate-deficient NTD model mice(n = 4) and control mice(n = 4). *Gapdh* was used as a loading control. *p < 0.05, **p < 0.01. **B** Analysis of KDM5A promoter-driven luciferase reporter activity in folate-deficient NE4C cells transfected with the control plasmid or the LEF1, PAX2, or TCF12 plasmid. The cells were harvested for the luciferase reporter assay after 48 h of transfection, n = 3, **p < 0.01. **C** PAX2 ChIP assays were performed using folate-deficient NE4C cells. The enrichment of PAX2 in the *Kdm5a* gene promoter was measured via RT‒qPCR. n = 3, **p < 0.01. **D** The PAX2 levels in folate-deficient NE4C cells was measured via Western blotting. GAPDH was used as a loading control. Protein levels were quantified using Image J software, n = 3. **E** RT‒qPCR analysis of *Pax2* mRNA and *Kdm5a* mRNA expression in NE4C cells after siPax2 transfection for 48 h. GAPDH was used as a loading control. n = 3, **p < 0.01. **F** Immunostaining for PAX2 and KDM5A in NE4C cells transfected with siPax2 for 24 h. Images were acquired with a confocal microscope, and nuclei were stained with DAPI. n = 4, ***p < 0.001. **G** Immunostaining for KDM5A and PAX2 in the neural tubes of folate-deficient NTD model embryos at E13.5(n = 6) and control embryos(n = 6). Images were acquired with a confocal microscope, and the nuclei were stained with DAPI. **H** IGV visualization showing the differential H3K4me3 peaks of *Pax2* promoters (2 kb upstream and downstream of the TSS) in NPSCs with folate deficiency or folate rescue. 10X FA: 40 mg/L synthetic folic acid supplementation. **I** H3K4me3 ChIP assays were performed on NPSCs with folate deficiency or folate rescue. Mouse IgG was used as a control. The enrichment of H3K4me3 in the promoters of the *Pax2* was measured via RT‒qPCR. n = 3, *p* < 0.05, **p < 0.01, ***p < 0.001. **J** The PAX2 levels in NPSCs with folate deficiency or folate rescue was measured via Western blotting. GAPDH was used as a loading control. Protein levels were quantified using Image J software. All the above data are presented as the means ± SEM from three biological replicates.

To investigate the mechanism underlying the downregulation of KDM5A transcription under conditions of folate deficiency, we screened for candidate transcription factor (TF)-binding motifs near the *KDM5A* promoter. As shown in JASPAR, TCF12, PAX2 and LEF1 binding motifs were enriched within the region 1 kilobase (kb) upstream of the *Kdm5a* promoter. We then evaluated the expression of these three genes in the brains and spines of folate-deficient E9.5 NTD model mice. RT‒qPCR analysis revealed that the mRNA expression of all three genes was downregulated (Fig. 7A), suggesting a positive regulatory relationship between these genes and *Kdm5a* under folate-deficient conditions. Furthermore, we sought to identify the specific TF that plays an important role in KDM5A transcription. A luciferase reporter assay revealed that only PAX2 significantly stimulated the luciferase activity driven by the KDM5A promoter (Fig. 7B). Moreover, ChIP‒qPCR using an anti-PAX2 antibody in NE4C cells revealed that PAX2 directly binds to the promoter of *KDM5A* and that this binding was attenuated under folate-deficient conditions compared with control conditions (Fig. 7C). WB analysis showed decreased PAX2 levels in folate-deficient NE4C (Fig. 7D). We next sought to determine whether the binding of PAX2 to *Kdm5a* was attenuated because of the decreased expression of *Kdm5a*. To this end, siRNA-mediated silencing of PAX2 was carried out to examine the effect on the level of *Kdm5a* transcription. In NE4C cells, *Kdm5a* mRNA expression decreased significantly with *Pax2* knockdown (Fig. 7E). The results of the immunofluorescence staining experiments were similar to those shown in Fig. 5E (Fig. 7F). Finally, we also observed via immunofluorescence staining that both the PAX2 and KDM5A protein levels were decreased in the folate-deficient NTD mouse model (Fig. 7G). Taken together, these results further revealed the underlying mechanism by which that folate deficiency regulates KDM5A expression through PAX2-mediated control of transcription.

To further investigate the mechanism by which folate downregulates PAX2-driven *Kdm5a* expression, we analysed H3K4me3 modifications on the *Pax2* promoter in NPSCs with or without folate. We observed significantly reduced H3K4me3 enrichment at the *Pax2* promoter in folate-deficient NPSCs compared to controls (Fig. 7H). This decrease was confirmed by ChIP-qPCR, and notably, folate supplementation restored H3K4me3 modification levels (Fig. 7I). Consistent with these findings, western blot analysis demonstrated reduced PAX2 protein expression in folate-deficient NPSCs, which was reversed upon folate supplementation (Fig. 7J). Collectively, these results imply folate deficiency epigenetically silences Pax2 expression via reduced H3K4me3 promoter modification, thereby impairing *Kdm5a* transcription.

### Decreased *KDM5A* mRNA expression and increased wnt target gene expression is correlated in NTD samples

On the basis of the association between the decreased KDM5A protein levesl during neurodevelopment and the increased expression of wnt target genes expression in the NTD mouse model, we sought to determine whether the levels of KDM5A, H3K4me3 or wnt target genes were also aberrant in NTD samples. Owing to the rapid decrease in the prevalence of NTDs, it was difficult to collect enough neural tissues from embryos from terminated pregnancies to measure the KDM5A protein level. As an alternative approach, we evaluated the mRNA levels of Wnt target genes in the brains of embryos with NTDs via NanoString analysis in 20 pairs low-folate samples and control samples.

**Fig. 8:**
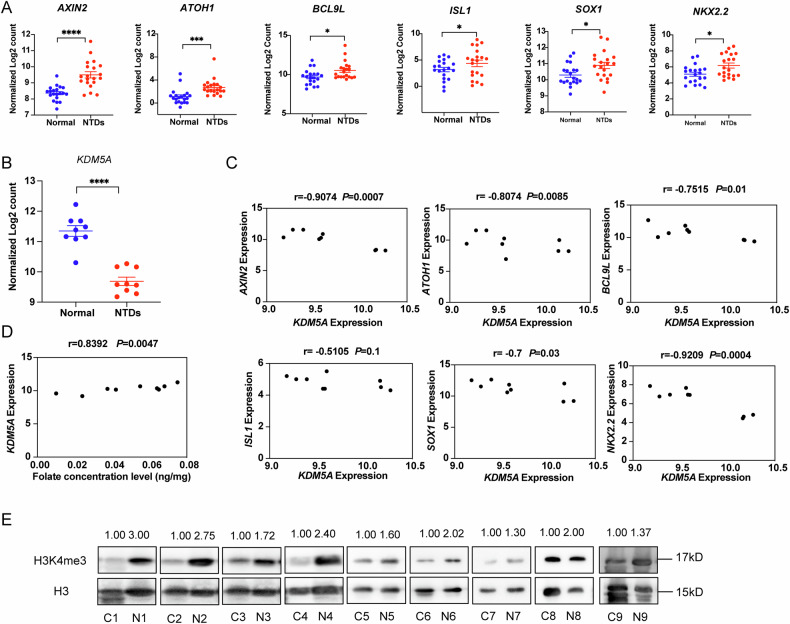
*KDM5A* gene expression was downregulated and H3K4me3 levels were increased in human foetuses with low-folate-induced NTDs. **A** mRNA expression levels of neurodevelopment-related genes in brain samples from foetuses with low-folate-induced NTDs, as determined by NanoString analysis (n = 20). **B** *KDM5A* mRNA expression in the brains of foetuses with low-folate-induced NTDs, as determined via NanoString analysis (n = 9). **C** Correlation analysis of the mRNA expression levels of *KDM5A* and neurodevelopment-related genes (n = 9). **D** Correlation analysis of the folate concentration and the *KDM5A* mRNA expression level (n = 9). **E** Increased H3K4me3 levels in brain samples from normal foetuses and foetuses with low-folate-induced NTDs were detected via western blotting (n = 9), C represents control and N represents NTDs. Fig. 8A and 8B are presented as the means ± SEM from three biological replicates.

First, we evaluated the expression of all six selected wnt/β-catenin pathway target genes, *AXIN2*, *ATOH1*, *BCL9L*, *ISL1*, *SOX1* and *NKX2.2* in NTD samples. NanoString analysis revealed that the expression of all six genes was significantly upregulated (Fig. 8A). Due to the scarcity of NTDs embryos brains, it was hardly to collect enough protein to check both KDM5A protein level and H3K4me3 level. Therefore, we chosen 9 pairs NTDs brain and control brains to evaluate the *KDM5A* mRNA level by NanoString first, the result showed it was significantly decreased (Fig. 8B). To further evaluate whether the *KDM5A* mRNA level is correlated with the mRNA levels of wnt target genes, we performed Pearson correlation analysis and found a significant negative correlation between the mRNA expression of *KDM5A* and that of each of the six wnt/β-catenin pathway target genes in these samples (Fig. 8C). In addition, we investigated the correlation between the *KDM5A* mRNA level and the folate concentration and found that the *KDM5A* mRNA level was significantly positively related to the folate concentration (Fig. 8D). We subsequently sought to determine whether H3K4me3 levels are altered in foetuses with low-folate-induced NTDs. WB analysis revealed that H3K4me3 levels were increased in 9 pairs of brain samples from nine anencephaly patients compared with brain samples from age- and sex-matched controls (Fig. 8E). Taken together, these results indicated that the *KDM5A* mRNA level was decreased but that H3K4me3 and wnt target gene levels were significantly increased. These findings further suggest that KDM5A modulates wnt/β-catenin signalling through H3K4me3 during the development of the nervous system in mammals in the context of folate deficiency. These results also suggest that KDM5A might have diagnostic potential and could be used as a predictive biomarker for NTDs.

## Discussion

Folate supplementation before and during pregnancy can prevent 30–70% of NTDs, but the preventive mechanism remains unclear. To date, several reports have demonstrated that wnt/β-catenin pathway activation leads to NTDs [31–33], and epigenetic mechanisms, such as histone modifications, are closely related to neural tube development [34–36]. Thus, defining additional mechanisms by which epigenetic factors contribute to wnt/β-catenin pathway activation is essential for understanding strategies for the treatment and prevention of NTDs. Our study identifying a novel epigenetic pathway wherein folate deficiency caues NTDs through the transcriptional silencing of the histone demethylase KDM5A, leading to H3K4me3-mediated hyperactivation of wnt/β-catenin signalling. We further delineate a upstream mechanism involving the folate-sensitive transcription factor PAX2, thereby revealing a regulatory axis that offers new nodes for potential therapeutic intervention.

Epigenetic dysregulation of wnt/β-catenin signalling genes has been well characterized in cancers, examples ranging from promoter hypermethylation of WNT10B [37] to histone modification-driven overexpression of WNT2 [38]. Our work extends this to developmental biology, demonstrating that a similar paradigm operates in folate-sensitive NTDs. We previously reported that significant Gcm1 overexpression promoted wnt/β-catenin signalling in patients with low-folate-induced NTDs via the formation of the Gcm1/β-catenin/TCF4 complex. However, a low folate concentration is not always accompanied by the overexpression of Gcm1 in NTDs. Therefore, wnt/β-catenin signalling activity may be increased via other mechanisms. Indeed, in neural progenitor cells, ASH2L loss was found to impair H3K4me3 modification and the transcriptional machinery specific for wnt/β-catenin signalling [39]. On the basis of the above findings, we hypothesized that histone mark represents a key point. By using RNA-seq and ChIP-seq analyses, we revealed that low folate induces a specific increase in H3K4me3 at wnt target gene promoters, resulting in pathway activation across multiple experimental models. This aligns with the established link between signalling pathways like PI3K/AKT [28] and H3K4 methylation, suggesting a broader network through which extracellular cues, including nutrient availability, can shape the epigenetic landscape.

Having established H3K4me3 as a key mediator, we investigated the responsible demethylase. Among the JARID family proteins, which are known to influence diverse physiology and disease states [40–42]. KDM5A, initially discovered as a retinoblastoma (RB)-binding protein, plays a critical role in cell growth, survival, angiogenesis, invasion, and migration and inhibits differentiation and senescence in cancer [26, 43, 44]. However, the function of KDM5A in neurodevelopment is very limited. Here, our data demonstrate that KDM5A, distinct from its homologs KDM5B/C, is the primary enzyme responsible for restraining H3K4me3 levels in the developing neural tube. The functional consequence of its loss was evident: siRNA-mediated knockdown of KDM5A sufficed to upregulate wnt target genes, and genetic ablation in mouse embryos and zebrafish led to consistent neurodevelopmental anomalies. This suggested KDM5A not merely as a general epigenetic modulator but as a dedicated safeguard against aberrant wnt pathway activation during neurulation.

A key upstream event in this cascade is the folate-dependent regulation of the transcription factor PAX2. Our combination of ChIP‒qPCR and luciferase assays confirmed that PAX2 directly activates *Kdm5a* transcription. Folate deficiency epigenetically silences PAX2 expression, at least in part through reduced H3K4me3 at its promoter, creating a positive feedback loop that amplifies the overall loss of demethylase activity. This finding is consistent with the established role of PAX2 in neurodevelopment and provides a mechanistic explanation for how a maternal diet low in folate is transduced into a specific epigenetic mark in the fetus. The restoration of PAX2 protein and amelioration of phenotypes upon folate treatment in our model highlight the dynamic nature of this regulation and the therapeutic potential of targeting this axis.

Collectively, our proposed model—that a low-folate maternal diet disrupts fetal H3K4me3 homeostasis via the PAX2-KDM5A axis, leading to wnt-driven NTDs. It resonates with the work of Lismer et al., who showed that paternal folate deficiency alters sperm H3K4me3 and increases birth defect risk [16]. We speculate that folate, as a methyl donor, may influence this pathway through multiple mechanisms, potentially including DNA methylation of the Pax2 locus, a compelling question for future studies aimed at understanding folate-preventable diseases like congenital heart disease and cleft lip and palate.

This study still has several limitations. For example, we did not use KDM5A-KO mice to demonstrate birth defects in offspring, for one reason embryonic lethality occurs in KDM5A homozygous knockout mice embryo, for another reason functional effects of KDM5A knockout are highly likely to be redundant with those of knockout of KDM5B or KDM5C. In addition, the number of clinical samples examined in this study was not sufficient for the analysis of nuclear KDM5A protein levels with the incidence of NTDs reducing. Our findings need to be confirmed in large patient cohorts before they can be translated into effective personalized screening approaches and therapeutic interventions. Taken together, our findings indicate that mechanisms of nongenetic inheritance mediated through maternal histone modifications are intimately involved in early mammalian development. These findings will also help to elucidate the mechanism by which folate deficiency causes NTDs or similar birth defects during the early stages of pregnancy.

### Materials

#### Cell culture and folate treatment

C57BL/6 mouse embryonic stem cells (C57mESCs), KM mouse embryonic fibroblasts (MEFs), NE4C cells and HEK293T cells were kindly provided by the Stem Cell Bank of the Chinese Academy of Sciences (Shanghai, China). sv129 mESCs were provided by Capital Institute of Pediatrics. Folate-deficient medium (Sigma‒Aldrich, USA) was prepared with a final concentration of 4 mg/L synthetic folic acid as a normal control. For culture under both normal and folate-deficient conditions, C57mESCs were seeded in culture dishes coated with MEFs. The normal cells were cultured with modified Eagle’s medium (Sigma‒Aldrich, USA) supplemented with 4 mg/L synthetic folic acid and 15% foetal bovine serum (FBS). The folate-deficient cells were cultured with MEM without folate supplemented with 15% FBS. The culture media for the C576mESCs, sv129 mESCs, and NE4C cells were changed daily. All the cells were maintained at 37 °C in a humidified atmosphere with 5% CO_2_ and passaged every 2–3 days. All cell lines were authenticated by STR profiling and tested as mycoplasma-free.

#### Western blot (WB) analysis

Proteins were extracted from cell lines and tissues with a nucleoprotein extraction kit (Sangon Biotech, China) or a total protein extraction kit (Sangon Biotech, China) following the manufacturer’s protocol. For Western blotting, proteins were separated via 10%-15% sodium dodecyl sulfate (SDS)–polyacrylamide gel electrophoresis and transferred to polyvinylidene difluoride membranes. Each membrane was incubated with primary antibodies at 4 °C overnight and was then immersed in 5% milk/PBST containing horseradish peroxidase (HRP)-conjugated secondary antibodies for 1 h. Bands were detected with Amersham™ ECL™ Prime Western Blotting Detection Reagent (GE Healthcare).

#### Real-time PCR (RT‒PCR)

Total RNA was isolated from cell lines, mouse tissue and zebrafish with TRIzol reagent (Invitrogen, USA). The total RNA from the various groups was converted into cDNA with TransScript First-Strand cDNA Synthesis SuperMix (TransGen Biotech, China). An UltraSYBR mixture (*CW*Biotech, China) was used for RT–PCR, and the relative mRNA levels in the different cell groups were quantified by analysis in a QuantStudio™ 7 Flex Real-Time PCR System (ABI, USA). The primers used in the related experiments are listed in Supplementary Table S1.

#### Measurement of folate concentrations

The folate concentrations in the cells and tissues were measured with a competitive receptor binding immunoassay kit (Chemiluminescent Immunoenzyme Assay, USA) and an Access 2 Immunoassay System (Beckman Coulter, USA). In brief, 10^6^ cells or 15 mg of brain tissue were collected in 1 ml of Tris buffer solution, subjected to 9 cycles of sonication, and centrifuged at 10000 rpm for 3 minutes at 4 °C before the folate concentrations in the supernatant were measured.

#### Chromatin Immunoprecipitation (ChIP), ChIP‒qPCR and ChIP‒seq

ChIP and ChIP-qPCR were performed with Enzymatic Chromatin IP Kit (Cell Signaling Technology, MA, USA) in accordance with the manufacturer’s protocol as our previously described [45]. Chromatin in DNA–protein complexes were analysed by qPCR with specific primers to amplify multiple regions of the genes. The percentage of DNA pulled down in the ChIP reaction (percent of input) was calculated as follows: Percent of input = 2% × 2(C[T] 2% input sample − C[T] IP sample), where C[T] = Ct = the PCR cycle threshold value. Each analysis included three independent ChIP experiments. Primers flanking specific regions were designed and are listed in Supplementary Table S2. Chromatin in DNA–protein complexes sequencing was conducted by Novogene (Beijing, China) our previously described [45]. ChIP-seq libraries were prepared using the KAPA HTP Library Preparation Kit complemented with NEXTflex DNA Barcodes from Bioo Scientific and performed by Novogene Corporation (Beijing, China). Subsequently, pair-end sequencing of sample was performed on Illumina platform (Illumina, CA, USA). Quality control was performed using FastQC (v0.12.1) with mean Q-score >30 across all bases. Index of the reference genome was built using BWA (v 0.7.12) and clean reads were aligned to the mm10 (GRCm38) reference genome using BWA mem (v 0.7.12). Differential Peaks were called using MACS2 (v2.2.7.1, q-value < 0.05), thereby obtaining information on DNA segments across the entire genome.

#### Luciferase assay

A TOP Flash construct (400 ng) and a Renilla reporter (400 ng) were separately mixed with a TK control reporter (50 ng) and cotransfected into KDM5A-knockout (KO) 293 T cells with Lipofectamine 3000. The cells in the positive control group were treated with LiCl for 12 h. After transfection for 48 h, luciferase activity was measured with a TOPFlash/FOPFlash assay. To this end, a Dual-Luciferase® Reporter Assay System (Promega, USA) was used to measure luciferase activity according to the manufacturer’s protocol in a Modulus Single Tube Multimode Reader (Promega, USA). For transfection of the pGL3-KDM5A luciferase reporter for KDM5A overexpression, 2 µg of pGL3-KDM5A was transfected with pCDNA3.1-Pax2, pCDNA3.1-LEF1 or pCDNA3.1-TCF12 into NE4C cells via Lipofectamine 3000. After 48 h, luciferase activity was measured as described above. Student’s *t* test was used to assess the statistical significance of differences.

#### Small interfering RNAs (siRNAs) and plasmid constructs

The siRNA sequences of mouse PAX2 (5′-CGCCCAGAAGATCTGAAACTAA-3′), human KDM5A (5′-ACUACCAAUGGAGGAUCUUAATT-3′) and mouse KDM5A (5′-GCACAATCCTATGACACTTGG-3′) were synthesized by Sangon (Shanghai, China). The cells were transfected with siRNAs with Lipofectamine 3000 according to the manufacturer’s instructions and were collected after 48 h of transfection. The human KDM5A overexpression plasmids were purchased from Addgene (Cat# 14799). The mouse PGL3-KDM5A, mouse pCDNA3.1-PAX2, mouse pCDNA3.1-TCF12, and mouse pCDNA3.1-LEF1 plasmids were constructed by Shenogen (Beijing, China).

#### Immunofluorescence staining

The cells and tissues were fixed with 4% paraformaldehyde. After permeabilization with 1% Triton X-100 for 15 minutes, the cells and tissues were blocked with an immunofluorescence blocking solution for 60 minutes and then incubated with a diluted monoclonal antibody overnight at 4 °C. The corresponding fluorescently labelled secondary antibody was then added, and the samples were incubated for 1 h at room temperature in the dark. 4’,6-Diamidino-2-phenylindole (DAPI) was added for nuclear staining at room temperature for 20 minutes, after which images were acquired under a fluorescence microscope (Zeiss LSM 710).

#### Generation of KDM5A-KO HEK293T cells

KDM5A-KO HEK293T cells were purchased from Beyotime (Shanghai, China). These KDM5A-KO HEK293T cells are polyclonal cells infected with lentivirus expressing the single guide RNA (sgRNA) and Cas9 and containing a puromycin resistance cassette and were characterized via a T7EI assay. The sgRNA sequence: TCTCCGCCAAAGGCCGGATG. The sequences of the PCR primers specific for KDM5A were as follows: Forwards 5′ CTTCAGCCAGCTTTTCTCCA 3′. Reverse 5′ TCAGTTCCCAAGTTCTAGGC3′. The cells were maintained in DMEM supplemented with 10% FBS and cultured at 37 °C with 5% CO_2_. This cell line was validated by short tandem repeat (STR) profiling and was free of mycoplasma contamination.

#### Differentiation of Folate-Free Induced Mouse Embryonic Stem Cells

C57BL/6 Mouse embryonic stem cells (mESCs) stably cultured in folate-free medium for six generations were maintained on a feeder layer of mouse embryonic fibroblasts (MEFs) with leukemia inhibitory factor (LIF, millpore, USA) supplementation for 48 hours (designated as Day 0). The cells were then digested using 0.25% trypsin, and residual MEFs were removed via differential adhesion. Subsequently, cells were resuspended at a density of 10⁵ cells/mL and seeded into culture dishes pre-coated with 1% agar in folate-free medium without LIF. The cells were subjected to suspension culture for 4 days, with half-medium replacement daily. By the end of this period, spherical cell aggregates (embryoid bodies, EBs) were visibly formed (designated as Day 4). The EBs were transferred to cell culture flasks pre-coated with 0.2% gelatin and further induced in folate-free medium supplemented with 1 μM retinoic acid (RA, Sigma, USA) for an additional 4 days to promote differentiation toward the neuroectodermal lineage (designated as Day 8). For folate rescue experiment, synthetic folic acid was supplemented at a concentration of 40 mg/L in the folate-free medium starting from Day 0. Cells harvested at Day 8 were subjected to Cut&Tag experiments.

#### NTDs mice model

NTDs mouse model were established as our previously described [45]. Composition of low-folate diet are following Casein (20%), Cystine (0.3%), Corn Starch (39.7%), Maltodextrin (13.2%), Sucrose (10%), Cellulose (5%), Soybean Oil (7%), Choline Bitartrate (0.25%), Mineral Mix S10022(3.5%), Vitamin Mix (1%) and Antioxidant (0.0014%). No specific randomization method was used. All procedures involving animal handling were performed according to the institutional guidelines approved by the Animal Ethics Committee of Shanxi Medical University. During the experiment, the mice were randomly grouped in accordance with the experiment design. The investigator was blinded to the group allocation of the mice. The sample size is described in the corresponding figure legend. No animals were excluded from the analysis.

#### Cleavage Under Targets and Tagmentation (Cut&Tag)

Cut&Tag was performed with Hyperactive Universal Cut&Tag Assay Kit (Vazyme, China) in accordance with the manufacturer’s protocol. For NTDs brains, the tissue was digested and then 100,000 cells were taken for subsequent experiments. The sequencing was conducted by Novogene (Beijing, China). In brief, tissues or cells were begin with utilizing antibody-guided targeting of the pA-Tn5 transposase to specific proteins, enabling precise tagmentation of adjacent DNA, followed by library amplification, sequencing, and bioinformatic analysis to map protein-genome interactions.

#### KDM5A lentivirus generation and infection

Recombinant lentivirus of mouse KDM5A were purchased from GenePharma (Shanghai, China). The lentivirus vector construct expressing short hairpin RNA (shRNA) complementary to the coding exon of the mice Kdm5a gene and tagged it with a green fluorescent protein (GFP). We generated 3 sh-Kdm5a sequences with1#(5′GCGGCTAGTAAGTAGCATTGA3′)2#(5′GCACAATCCTATGACACTTGG3′) and 3#(5′GCACAGTATGGCAAATTTAGA3′) respectively. There was also a non-silencing shRNA control with GFP created. All the lentiviruses were packaging with titer 1×10⁹ TU/mL). For the KDM5A lentivirus infection, the lentiviral vectors were transfected into 293 T cells with the sh-NC and sh-KDM5A packaging plasmid mix in serum-free Opti-MEM using the lipofectamine 3000 reagent with multiplicity of infection (MOI) of 20 and with or without 5 μg/ml polybrene. After infection for 48 hours, the cells were harvested for protein extracted and western blot.

#### Embryo dissection and whole embryo culture (WEC)

Pregnant females were euthanized at E8.5 by cervical dislocation, and uteri were transferred to pre-warmed dissection medium. Dissections were performed under a stereomicroscope (Nikon SMZ1270) at 37°C in phenol red-free DMEM/F12 (Procell, 25×2030) supplemented with 10% FBS (Procell, 164210-50), 3 mg/ml D-glucose, and 1% penicillin-streptomycin (Solarbio, P1400). Embryos at the 4-6 somite stage with intact yolk sacs were selected for experiments [46].

Selected embryos were cultured in a rotating bottle system (WEC001-S, Beijing Liangyi Biotechnology) using heat-inactivated (56°C, 30 min) rat serum supplemented with 2 mM GlutaMAX (Solarbio, G0200), antibiotics, 11 mM HEPES (Solarbio, H1095), and 3 mg/mL D-glucose. Three embryos per bottle were cultured at 37°C with continuous rotation (15 rpm) under 20% O₂, 5% CO₂, and 75% N₂ for the first 24 hours, followed by 40% O₂ for the remaining culture period after medium replacement. Total culture duration was 48 hours, reaching the equivalent of E10.5.

#### Lentiviral microinjection

Microinjection procedures were adapted from a previously published method [47]. Freshly dissected E8.5 embryos were microinjected with lentiviral particles targeting KDM5A (sh-KDM5A constructs KDM5A-3, titer ~1×10⁹ TU/mL) or control GFP lentivirus (sh-NC; titer ~1×10⁹ TU/mL). Borosilicate glass capillaries (Sutter Instrument, BF100-78-10) were pulled using a programmable micropipette puller (Sutter P-97) to generate injection needles with optimal tip dimensions. Microinjections were performed under stereomicroscopic guidance using a micromanipulator-mounted FemtoJet 4x (Eppendorf). Approximately 100 nL of viral suspension was injected into the amniotic cavity of each embryo before transfer to the WEC system.

#### Embryo analysis

After 48 hours of culture, embryos were harvested and evaluated. Transduction efficiency was assessed by GFP fluorescence visualization using a Leica Thunder Imager M205FA stereomicroscope. Neural tube closure was systematically assessed at multiple levels, and overall embryonic development was evaluated using the standardized post-implantation embryo scoring system by investigators blinded to experimental conditions [46]. For subsequent analyses, embryos were flash-frozen for protein/RNA extraction. All experiments included at least three biological replicates.

#### Zebrafish studies

Zebrafish embryos were obtained by mating adult fish via standard methods. All the fish strains were maintained individually as inbred lines. The wild-type zebrafish strain was provided by the China Zebrafish Resource Center. The embryos were reared at 28.5 °C until processing at the desired stages for analyses. All zebrafish experiments were performed in strict accordance with the Regulations for the Care and Use of Laboratory Animals published by the Ministry of Science and Technology of China and the Institute of Zoology’s Guidelines for the Care and Use of Laboratory Animals. The embryo stages were determined via morphological evaluation as previously described [48].

#### CRISPR/Cas9 genome editing and embryo injection

For KDM5A knockout, zebrafish embryo yolk was injected with the KDM5A sgRNA and Cas9 plasmid. At 24 hours postfertilization (hpf), the embryos were harvested for in situ hybridization (ISH). The KDM5A sgRNA sequence (5’-TGTGATGAAGAAGGTGGCCCCGG-3’) was designed via the CRISPRscan website and synthesized by Sangon (Shanghai, China).

#### In situ hybridization (ISH)

Digoxigenin-UTP-labeled RNA probes for Axin2, Atoh1a, Sox1b, Nkx2.2b, ISL1 were synthesized in vitro from linearized DNA templates using the T7 RNA Polymerase, system (Roche, Cat#10881775001) following the manufacturer’s protocol. ISH was conducted according to standard procedures as previously described [49, 50]. Representative images were taken using an Olympus IX-71 microscope. Details of the zebrafish probe primer are provided in Supplementary Table S3.

#### RNA-seq

RNA sequencing was conducted by Annoroad Gene Technology (Beijing, China). For each group, 40–50 pooled embryos at 24 hpf were snap-frozen in liquid nitrogen and stored at −80 °C. Total RNA was extracted using Trizol (Invitrogen, USA). RNA purity was verified with the kaiaoK5500® Spectrophotometer (Kaiao, Beijing, China), while integrity and concentration were assessed using the RNA Nano 6000 Assay. Each sample used 2 μg of RNA as input material for library preparation.

Libraries were constructed with the NEBNext® Ultra™ RNA Library Prep Kit for Illumina® (#E7530L, NEB, USA) per the manufacturer’s guidelines, with index codes for sample identification. Clustering was conducted on a cBot system with HiSeq PE Cluster Kit v4-cBot-HS (Illumina), and 150 bp paired-end reads were generated on an Illumina platform.

Quality control was performed with FastQC (v0.11.5), and low-quality reads were filtered with NGSQC (v2.3.3). Clean reads were aligned to the reference genome (http://asia.ensembl.org/index.html) using HISAT2 (v2.1.0) under default parameters. Gene expression was quantified with StringTie (v1.3.3b), and differential expression analysis was conducted with DESeq (v1.28.0). DEGs were identified with a fold change of ≥2 (|log2FC | ≥ 1) and significance level of p ≤ 0.05, adjusted by the Benjamini-Hochberg FDR method. DEG annotations were based on ENSEMBL, NCBI, UniProt, GO, and KEGG databases.

#### Human samples

All clinical samples used in this study were obtained from the city of Lvliang in Shanxi Province in northern China with informed consent from the parents. The enrolled pregnant women were diagnosed by trained clinicians via ultrasonography. The surgical procedures were performed as previously described [51]. The epidemiological method was described in detail in our previous publication [52]. The Ethics Board of the Capital Institute of Pediatrics approved the study protocol. Sample size of human NTDs samples was chosen based on G-power calculation. The information about the clinical samples is listed in Supplementary Table S4.

#### NanoString analysis

NanoString analyses were performed as previously described [45]. The raw counts were normalized to the internal transcript abundances for five reference genes, namely, ACTB, TUBB, CLTC, TBP and POLR1B. Quality control and analysis of the raw NanoString data were conducted with nSolver Analysis Software v4.0 (NanoString Technologies, USA). Student’s t test was used to compare normalized expression values between normal control samples and NTD samples.

#### Antibodies and reagents

The primary antibodies used in this study and their sources were as follows: anti-KDM5A (CST, #D28B10), anti-H3K4me3 (CST, #C42D8), anti-Pax2 (CST, #9666), anti-histone H3 (Abcam, #14269), and anti-GAPDH (Abcam, #ab8245). HRP-conjugated and fluorescent secondary antibodies were obtained from ZSBIO. Folate (4 mg/L, Sigma‒Aldrich) was diluted with low-glucose DMEM (Sigma‒Aldrich) supplemented with 3.7% filtered sodium bicarbonate (Beijing Chemical Reagent Company) and UltraPure™ distilled water (Thermo). MTX (0.12 µM, Sigma‒Aldrich) was diluted in DMSO (Sigma‒Aldrich).

#### Statistical analysis

All experiments were repeated at least three times with consistent results. All the data are presented as the means ± SEMs. Statistical analyses were performed with two-tailed Student’s t test for comparisons between two groups or with ANOVA followed by Tukey’s post hoc test for comparisons among more than two groups after verifying the normality of the data. Statistically significant differences are indicated as follows: *P < 0.05, **P < 0.01, ***P < 0.001, and ****P < 0.0001. All the statistical analyses were performed with SPSS 22.0 software. Variance is similar between the groups that are being statistically compared. Investigators were blinded to the group allocation during the experiment and when assessing the outcome in all experiments including animal experiments.

## Supplementary information

supplementary tables and figure legends

Supplementary Table S1

Supplementary Table S2

Supplementary Table S3

Supplementary Table S4

Figure S1

Figure S2

Figure S3

Figure S4

Figure S5

Figure S6

uncropped WB image

## Data Availability

All relevant data are available within the article and its supplementary information files or can be obtained from the corresponding author upon reasonable request. The ChIP-seq and Cut&Tag data have been deposited in the GEO database under accession number GSE225567, GSE299191. The Cut&tag for H3K4me3 in neural progenitor cells differentied folate-free mESC has been deposited in the Genome Sequence Archive in National Genomics Data Center, China National Center for Bioinformation / Beijing Institute of Genomics, Chinese Academy of Sciences (GSA: CRA031711) that are publicly accessible at https://ngdc.cncb.ac.cn/gsa.The RNA-seq data have been deposited in the GEO database under accession number GSE280830.
