# Supplementary material for: Loss of KDM5A-mediated H3K4me3 demethylation promotes aberrant neural development by Wnt/β-catenin pathway activation
Source: Cell Death Dis. 2025 Nov 20;16(1):853. doi: 10.1038/s41419-025-08208-5 (PMC12644828; doi:10.1038/s41419-025-08208-5)
Supplement: Supplementary file 1 — supplementary tables and figure legends [file 41419_2025_8208_MOESM1_ESM.docx]

# Tables

Supplementary Table S1: Primers used for Real Time RT-PCR

Supplementary Table S2: Primers used for ChIP-qPCR

Supplementary Table S3: Primers used for zebrafish probe

Supplementary Table S4: Clinical manifestations of normal fetus and NTDs fetus

# Supplementary Figure legends

**Figure S1: Increased H3K4me3 induced by folate deficiency binding neurodevelopment genes promoters in wnt/catenin pathway**.

**(A)** RT-qPCR of neurodevelopment-related genes *Axin2、Bcl9l、Atoh1、Nkx2.2、Sox1* and *Isl1* gene in NE4C with folate deficiency. All the values were normalized to *Gapdh* at mRNA level in the same sample. n=3, **p* < 0.05, ***p* <0.01. **(B)** RT-qPCR of neurodevelopment-related genes *Axin2、Bcl9l、Atoh1、Nkx2.2、Sox1* and *Isl1* gene in sv129 mESC with 0.12uM MTX for 24h or with 40mg/L synthetic folic acid rescue for 6h. All the values were normalized to GAPDH at mRNA level in the same sample. n=3, **p* < 0.05, ***p* <0.01, ****p* <0.001. All the above data are the mean ± SEM from three biological replicates.

**Figure S2: KDM5A is required for upregulation of neurodevelopment-related genes in wnt/catenin pathway under folate deficiency.**

**(A)** KDM5A level in C57mESC and 293T with folate deficiency was measured by Western Blot, respectively. GAPDH were used as loading control. n=3. **(B)** KDM5B level in C57mESC and 293T with folate deficiency was measured by Western Blot, respectively. GAPDH were used as loading control. n=3. **(C)** Morphology and crystal violet staining of folate-deficient 293T are observed with an optical microscope at magnifications of 5x and 10x respectively, n=3. **(D)** Folate concentration in folate deficient 293T was measured by ACSSII, n=9, ****p* <0.001. **(E)** *Kdm5a* mRNA in folate deficient 293T. The value was normalized to *Gapdh* at mRNA level in the same sample, n=3, **p* < 0.05. **(F)** KDM5A and H3K4me3 level in folate deficient 293T was measured by Western Blot. GAPDH and H3 were used as loading control separately, n=3. **(G)** KDM5A and H3K4me3 level in 293T was measured by Western Blot, after KDM5A over expression and siKDM5A transfection with or without folate, n=3. GAPDH and H3 were used as loading control separately, n=3. All the above data are the mean ± SEM from three biological replicates.

**Figure S3: H3K4me3 enriched in wnt signaling pathway and neurodevelopment genes with KDM5A knockout.**

**(A)** The efficiency of KDM5A knockout with CRISPER/Cas9 by DNA agarose electrophoresis with T7 Endonuclease I (T7E1), the lane adjacent to the marker exhibits distinct alterations in band intensity compared to the first three lanes, suggesting potential cleavage or smearing. **(B)** Schematic of C57mESC differentiating the neuronal progenitors with folate-deficiency. RA：Retinoic acid；LIF: Leukemia inhibitory factor. EB：Embryoid body. NPSCs: Neural progenitor stem cells.

**Figure S4: Folate deficiency caused induced levels of H3K4me3 in the NTDs animal model.**

**(A)** HE staining was performed on E13.5d NTDs mouse brain(n=6) and control mouse brain(n=6). Representative images were shown. Scale bar, 1mm. **(B)** GO enrichment analysis of different H3K4me3 peaks upon E9.5d mouse NTDs brains. **(C)** RT-qPCR analysis of neurodevelopment-related genes mRNA in 9.5day folate deficient NTDs spine. *Gapdh* were used as loading control, **p* < 0.05, ***p* <0.01, ****p* <0.001. Fig.S4C are the mean ± SEM from three biological replicates.

**Figure S5: KDM5A knockdown with lentivirus in 293T cell**

**(A)** Immunostaining of 293T with sh-KDM5A for 48 hours, the image was obtained in bright field、KDM5A lentiviral and KDM5A lentiviral with polybrene using a Leica Thunder Imager M205FA stereomicroscope. n=3. Scale bar, 1mm. **(B)** KDM5A levels in the 293T cell with sh-KDM5A for 48h were measured via Western blotting. GAPDH were used as loading controls for KDM5A. 1#、2# and 3# was referred sh-KDM5A sequence1、2、3 respectively，P was represented for polybrene. n=3.

**Figure S6: Wnt targets genes were upregulated in KDM5A-KO zebrafish**

**(A)** Volcano plot of different genes in 24hpf zebrafish upon KDM5A knockout with CRISPR/cas9. The x-axis shows the differential changes of gene expression; the expression is up (yellow) and down(blue). The y-axis shows the number of differential of genes. **(B)** RT-qPCR analysis of neurodevelopment-related genes mRNA in 24hpf zebrafish embryos with KDM5A knockdown with CRISPR/Cas9(n=40) and wide type（WT，n=50）. *β-actin* were used as loading control. **p* < 0.05, ***p* <0.01, ****p* <0.001. Data are presented as the means ± SEM from three biological replicates.
