# Supplementary material for: Loss of KDM5A-mediated H3K4me3 demethylation promotes aberrant neural development by Wnt/β-catenin pathway activation
Source: Cell Death Dis. 2025 Nov 20;16(1):853. doi: 10.1038/s41419-025-08208-5 (PMC12644828; doi:10.1038/s41419-025-08208-5)
Supplement: Supplementary file 2 — Supplementary Table S1 [file 41419_2025_8208_MOESM2_ESM.docx]

Supplementary Table S1: Primers used for Real Time RT-PCR

| Name | Forward/  Reverse | Sequence(5'to3') | Category |
| --- | --- | --- | --- |
| Mouse-GAPDH | Forward | TGACCTCAACTACATGGTCTACA | RT-PCR |
|  | Reverse | CTTCCCATTCTCGGCCTTG | RT-PCR |
| Human-GAPDH | Forward | TGTGGGCATCAATGGATTTGG | RT-PCR |
|  | Reverse | CCCTCCAGGGGATCTGTTTG | RT-PCR |
| Human-KDM5A | Forward | AGCCGAGTTGGGAGGAGTT | RT-PCR |
|  | Reverse | TGGACTCTTGGAGTGAAACGA | RT-PCR |
| Mouse-KDM5A | Forward | CACAGACCCGCTGAGTTTTAT | RT-PCR |
|  | Reverse | CTTCACAGGCAAATGGAGGTT | RT-PCR |
| Mouse-Axin2 | Forward | TGACTCTCCTTCCAGATCCCA | RT-PCR |
|  | Reverse | TGCCCACACTAGGCTGACA | RT-PCR |
| Mouse-Bcl9l | Forward | AGCAGCACCTAATGGGCAAAG | RT-PCR |
|  | Reverse | GGATAAGTCGAACTCAGGAATGC | RT-PCR |
| Mouse-Atoh1 | Forward | GAGTGGGCTGAGGTAAAAGAGT | RT-PCR |
|  | Reverse | GGTCGGTGCTATCCAGGAG | RT-PCR |
| Mouse-Nkx2.2 | Forward | AAGCATTTCAAAACCGACGGA | RT-PCR |
|  | Reverse | CCTCAAATCCACAGATGACCAGA | RT-PCR |
| Mouse-Sox1 | Forward | GATGCCACCAACGCTAAAGC | RT-PCR |
|  | Reverse | TTGCGGTTGAAGTCCAGGC | RT-PCR |
| Mouse-ISL1 | Forward | ATGATGGTGGTTTACAGGCTAAC | RT-PCR |
|  | Reverse | TCGATGCTACTTCACTGCCAG | RT-PCR |
| Mouse-TCF12 | Forward | ATGTACTGTGCTTATCCTGTCCC | RT-PCR |
|  | Reverse | GGTGCATATACCGTTTTCCCATT | RT-PCR |
| Mouse-Pax2 | Forward | AAGCCCGGAGTGATTGGTG | RT-PCR |
|  | Reverse | CAGGCGAACATAGTCGGGTT | RT-PCR |
| Mouse-LEF1 | Forward | TGTTTATCCCATCACGGGTGG | RT-PCR |
|  | Reverse | CATGGAAGTGTCGCCTGACAG | RT-PCR |
| Zebrafish-Axin2 | Forward | AATGGCAGTTCAGCATTTCC | RT-PCR |
|  | Reverse | CTCGAACACAGCACCACACT | RT-PCR |
| Zebrafish-Bcl9l | Forward | CGGTGATGAATGGTGTTCAG | RT-PCR |
|  | Reverse | AGCTAGGCTGGTGGTGAAGA | RT-PCR |
| Zebrafish-Atoh1 | Forward | TGGAATGAGCACGGATACAA | RT-PCR |
|  | Reverse | GCTGCTCTTCCTGAAGTTGG | RT-PCR |
| Zebrafish-Nkx2.2 | Forward | GACATTTTGGACCTCCCTGA | RT-PCR |
|  | Reverse | GGACAGGCCGTGTAATGAGT | RT-PCR |
| Zebrafish-Sox1 | Forward | ACCGATCTCCTGTTCCAGTG | RT-PCR |
|  | Reverse | GAGAGTGCAAGTCCGTCTCC | RT-PCR |
| Zebrafish-ISL1 | Forward | GACAAGAGTGGCCCATCAAT | RT-PCR |
|  | Reverse | CCAGTACGAGGACGGAACAT | RT-PCR |
| Zebrafish-*β-actin* | Forward | ATGGATGATGAAATTGCCGCAC | RT-PCR |
|  | Reverse | ACCATCACCAGAGTCCATCACG | RT-PCR |
