# Supplementary material for: Loss of KDM5A-mediated H3K4me3 demethylation promotes aberrant neural development by Wnt/β-catenin pathway activation
Source: Cell Death Dis. 2025 Nov 20;16(1):853. doi: 10.1038/s41419-025-08208-5 (PMC12644828; doi:10.1038/s41419-025-08208-5)
Supplement: Supplementary file 3 — Supplementary Table S2 [file 41419_2025_8208_MOESM3_ESM.docx]

Supplementary Table S2: Primers used for ChIP-qPCR

| Name | | Forward/  Reverse | | Sequence(5'to3') | | Category |  |
| --- | --- | --- | --- | --- | --- | --- | --- |
|  | | Reverse | | CTTCACAGGCAAATGGAGGTT | | ChIP-qPCR |  |
| Mouse-Axin2 | | Forward | | GGCTGCTGGACTTTGGTTAG | | ChIP-qPCR |  |
|  | | Reverse | | TGCTCAGGTTAGGCAAACAA | | ChIP-qPCR |  |
| Mouse-Bcl9l | | Forward | | GGAAGGGAAAAGGTCTGAGG | | ChIP-qPCR |  |
|  | | Reverse | | ACTCCCTCAGCCTCCTGTCT | | ChIP-qPCR |  |
| Mouse-Atoh1 | | Forward | | ACTCCCTCAGCCTCCTGTCT | | ChIP-qPCR |  |
|  | | Reverse | | CTTCTGTTGAGCGTTTGCTG | | ChIP-qPCR |  |
| Mouse-Nkx2.2 | | Forward | | GGACCAGGGCTAGACATTGA | | ChIP-qPCR |  |
|  | | Reverse | | CTCAGAGCACAGTGGGGACT | | ChIP-qPCR |  |
| Mouse-Sox1 | | Forward | | ACTGGGCTTTGGGGTTATGT | | ChIP-qPCR |  |
|  | | Reverse | | AAGCGGTATGGTCAAACACC | | ChIP-qPCR |  |
| Mouse-ISL1 | | Forward | | ATGCCCTCTTCTGACTTCCA | | ChIP-qPCR |  |
|  | | Reverse | | GTAGGTTCCCAGTGTCGTGA | | ChIP-qPCR |  |
| Mouse-KDM5A | | Forward | | TCGCTTCTCCGTGACCAATA | | ChIP-qPCR |  |
|  |  | |  | |  | | |
