# Supplementary material for: Loss of KDM5A-mediated H3K4me3 demethylation promotes aberrant neural development by Wnt/β-catenin pathway activation
Source: Cell Death Dis. 2025 Nov 20;16(1):853. doi: 10.1038/s41419-025-08208-5 (PMC12644828; doi:10.1038/s41419-025-08208-5)
Supplement: Supplementary file 4 — Supplementary Table S3 [file 41419_2025_8208_MOESM4_ESM.docx]

Supplementary Table S3: Primers used for Zebrafish ISH Probe

| Name | | Forward/  Reverse | | Sequence(5'to3') | | Category |  |
| --- | --- | --- | --- | --- | --- | --- | --- |
|  | | Reverse | | CTCGAACACAGCACCACACT | | ISH Probe |  |
| Zebrafish-Axin2 | | Forward | | AATGGCAGTTCAGCATTTCC | | ISH Probe |  |
|  | | Reverse | | AGCTAGGCTGGTGGTGAAGA | | ISH Probe |  |
| Zebrafish-Bcl9l | | Forward | | CGGTGATGAATGGTGTTCAG | | ISH Probe |  |
|  | | Reverse | | GCTGCTCTTCCTGAAGTTGG | | ISH Probe |  |
| Zebrafish-Atoh1a | | Forward | | TGGAATGAGCACGGATACAA | | ISH Probe |  |
|  | | Reverse | | GGACAGGCCGTGTAATGAGT | | ISH Probe |  |
| Zebrafish -Nkx2.2a | | Forward | | GACATTTTGGACCTCCCTGA | | ISH Probe |  |
|  | | Reverse | | GGACAGGCCGTGTAATGAGT | | ISH Probe |  |
| Zebrafish -Sox1b | | Forward | | CTGCATGGGGGGATGAACC | | ISH Probe |  |
|  | | Reverse | | CAGCATCTGAGGCAGGCACC | | ISH Probe |  |
| Zebrafish -ISL1 | | Forward | | AGCCTGCTTTCCAACAACTG | | ISH Probe |  |
|  | | Reverse | | GGGAAAAGTGTGGAGGGGTA | | ISH Probe |  |
|  |  | |  | |  | | |
