# Supplementary material for: Loss of KDM5A-mediated H3K4me3 demethylation promotes aberrant neural development by Wnt/β-catenin pathway activation
Source: Cell Death Dis. 2025 Nov 20;16(1):853. doi: 10.1038/s41419-025-08208-5 (PMC12644828; doi:10.1038/s41419-025-08208-5)
Supplement: Supplementary file 5 — Supplementary Table S4 [file 41419_2025_8208_MOESM5_ESM.docx]

Supplementary Table S4： Clinical manifestations of normal fetus and NTD fetus

| Sample type | 编号 | tissue | Gender | Gestational Weeks |
| --- | --- | --- | --- | --- |
| Normal | A1910 | Brain | female | 22 |
| Normal | A2715 | Brain | female | 36 |
| Normal | A1385 | Brain | female | 26 |
| Normal | A1581 | Brain | male | 21 |
| Normal | A1490 | Brain | male | 25 |
| Normal | A2570 | Brain | female | 22 |
| Normal | A2499 | Brain | male | 26 |
| Normal | A2542 | Brain | female | 25 |
| Normal | A2390 | Brain | male | 18 |
| Normal | A1462 | Brain | female | 15 |
| Normal | A2415 | Brain | male | 23 |
| Normal | A1753 | Brain | male | 16 |
| Normal | A2633 | Brain | female | 24 |
| Normal | A2664 | Brain | female | 22 |
| Normal | A2625 | Brain | female | 23 |
| Normal | A2550 | Brain | male | 24 |
| Normal | A2630 | Brain | female | 24 |
| Normal | A2853 | Brain | male | 27 |
| Normal | A2867 | Brain | male | 24 |
| Normal | A2628 | Brain | female | 25 |
| NTDs | A2515 | Brain | female | 20 |
| NTDs | A2545 | Brain | female | 33 |
| NTDs | A1589 | Brain | female | 27 |
| NTDs | A1520 | Brain | male | 22 |
| NTDs | A2620 | Brain | male | 16 |
| NTDs | A1529 | Brain | female | 21 |
| NTDs | A1345 | Brain | male | 19 |
| NTDs | A1272 | Brain | female | 23 |
| NTDs | A1676 | Brain | female | 24 |
| NTDs | A2190 | Brain | female | 19 |
| NTDs | A2289 | Brain | male | 16 |
| NTDs | A2364 | Brain | male | 21 |
| NTDs | A2495 | Brain | female | 15 |
| NTDs | A2618 | Brain | male | 18 |
| NTDs | A1446 | Brain | female | 20 |
| NTDs | A2248 | Brain | female | 26 |
| NTDs | A1447 | Brain | female | 24 |
| NTDs | A1504 | Brain | male | 22 |
| NTDs | A2037 | Brain | male | 20 |
| NTDs | A1677 | Brain | female | 21 |
