# Supplementary figures and images for: Loss of KDM5A-mediated H3K4me3 demethylation promotes aberrant neural development by Wnt/β-catenin pathway activation
Source: Cell Death Dis. 2025 Nov 20;16(1):853. doi: 10.1038/s41419-025-08208-5 (PMC12644828; doi:10.1038/s41419-025-08208-5)

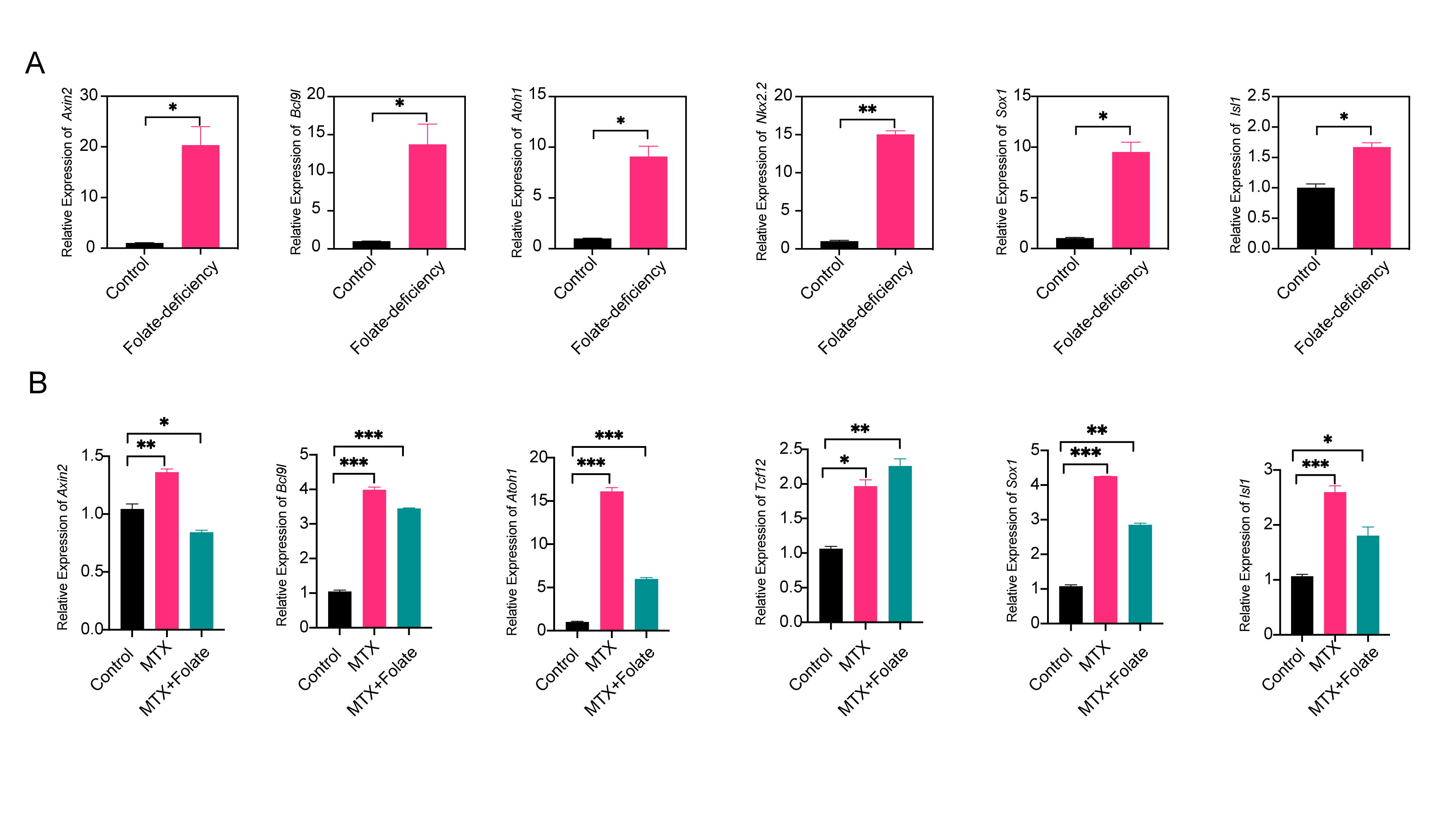

Supplement: Supplementary file 6 — Figure S1 [file 41419_2025_8208_MOESM6_ESM.tif]

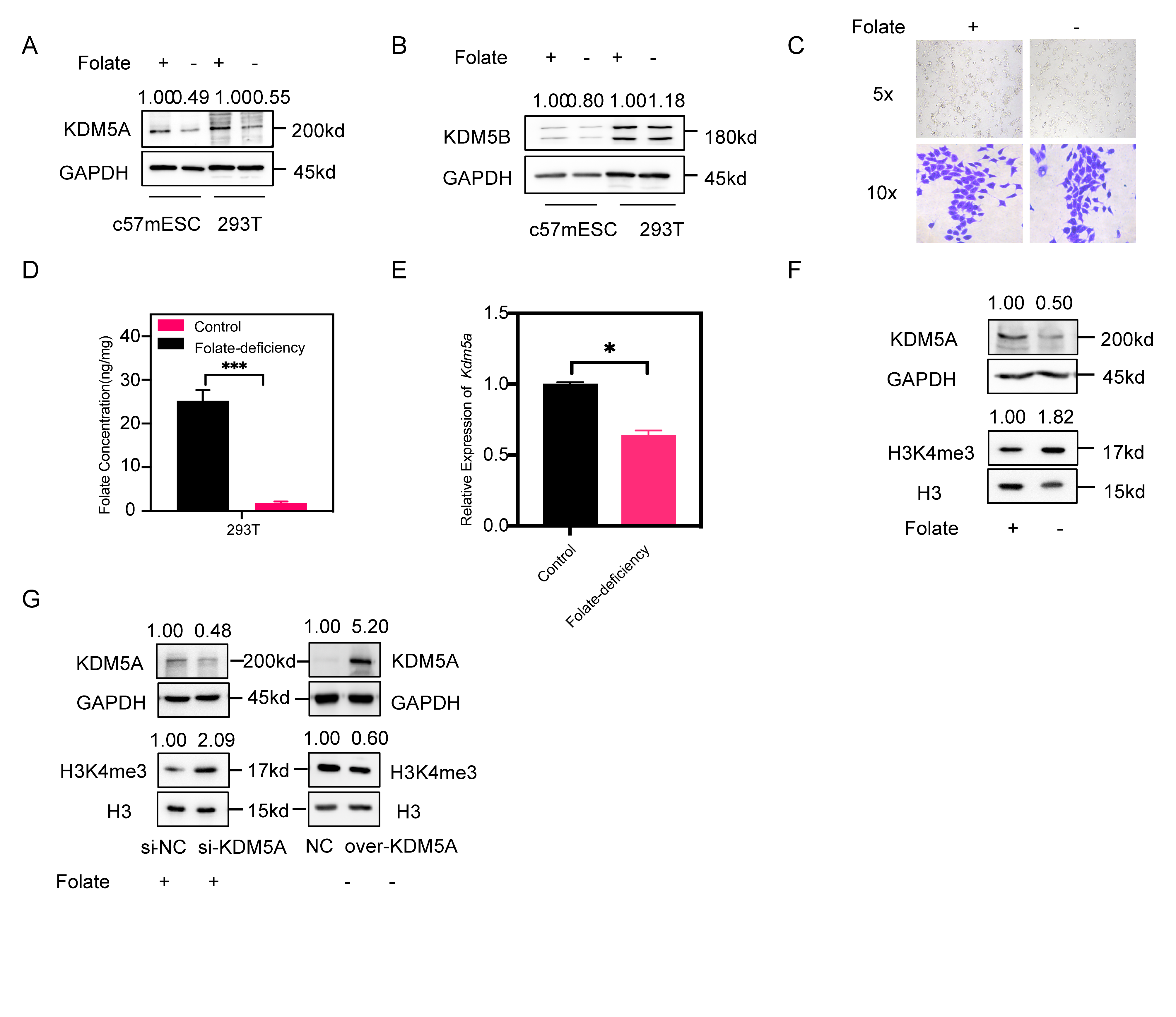

Supplement: Supplementary file 7 — Figure S2 [file 41419_2025_8208_MOESM7_ESM.tif]

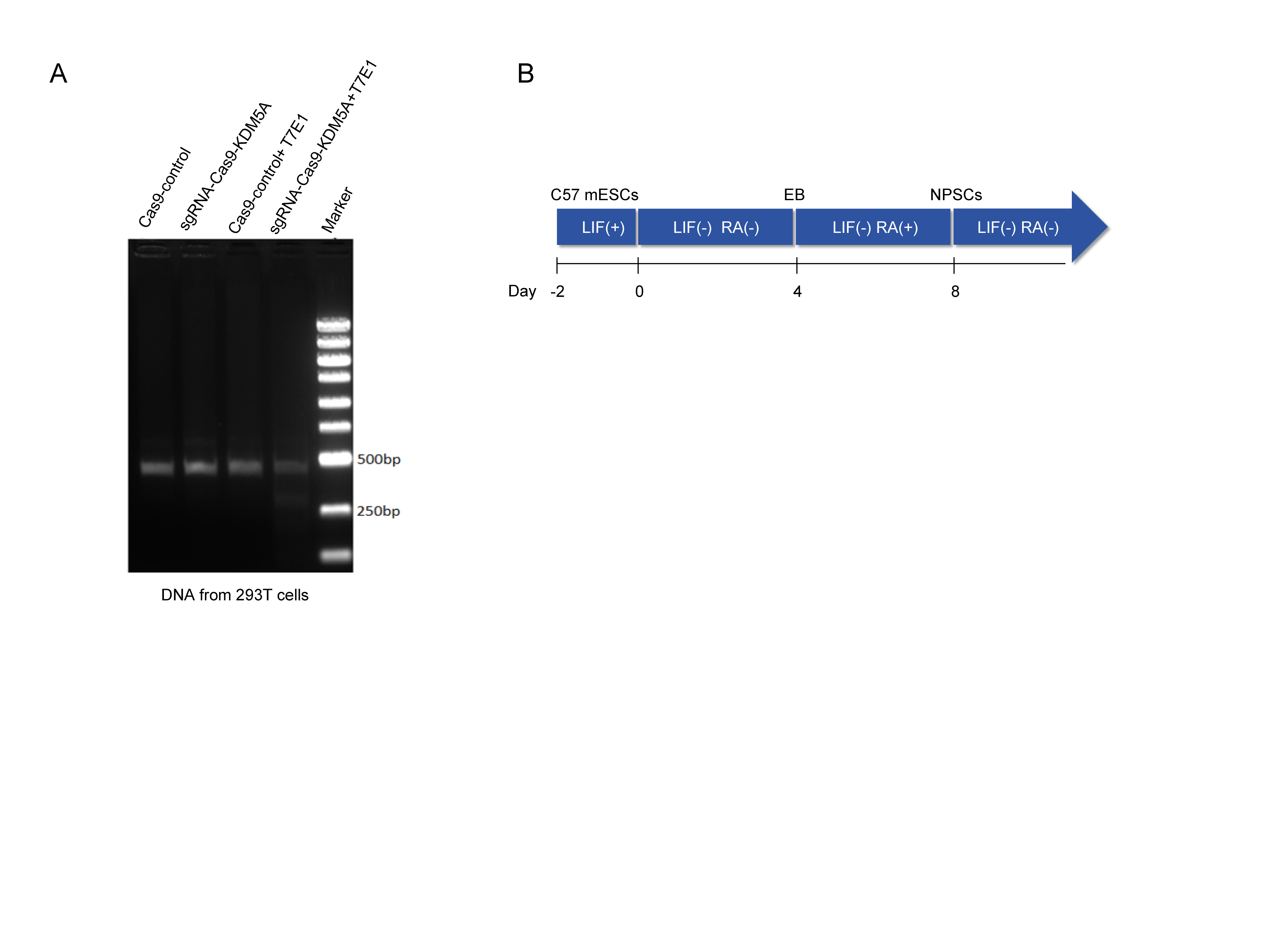

Supplement: Supplementary file 8 — Figure S3 [file 41419_2025_8208_MOESM8_ESM.tif]

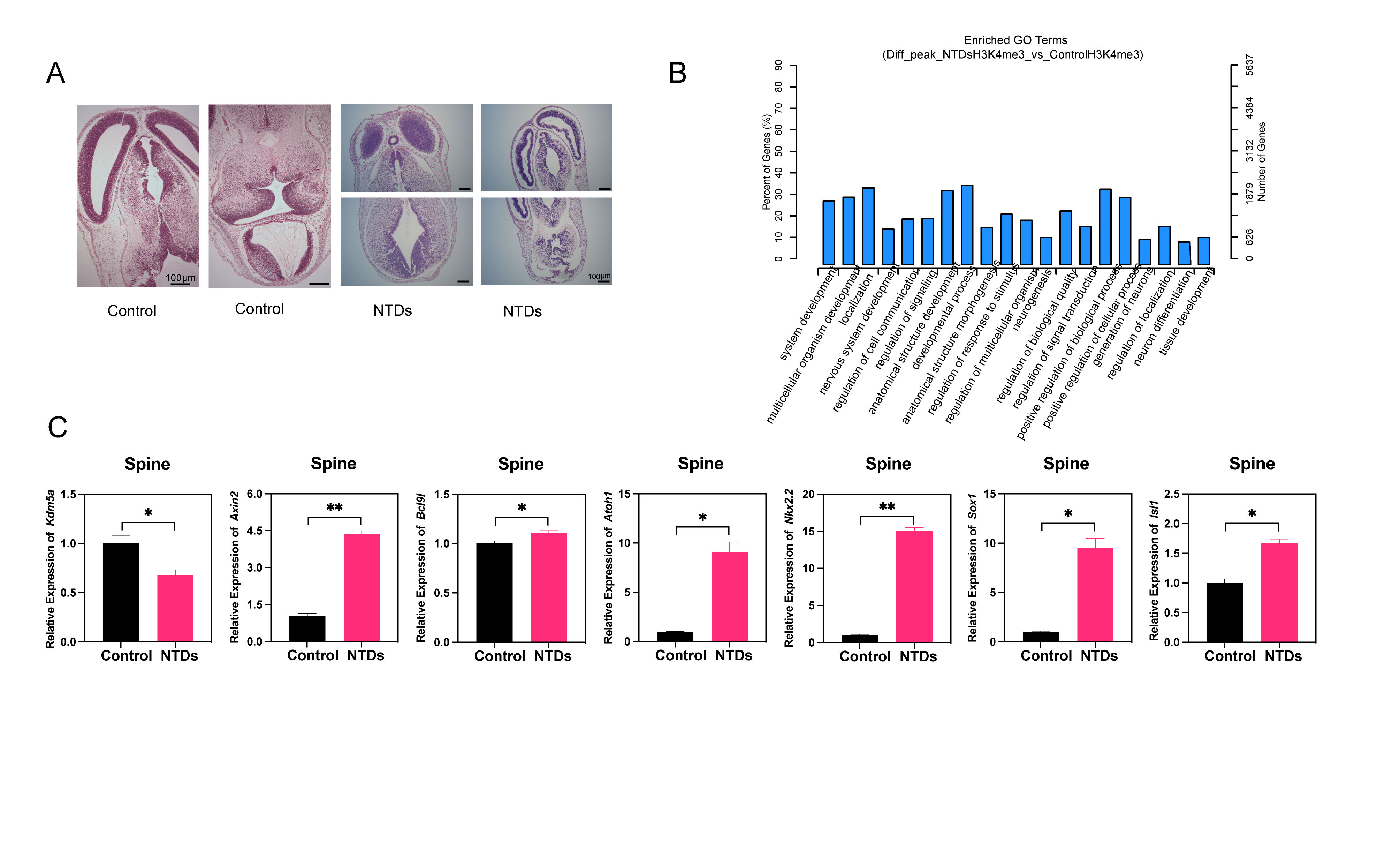

Supplement: Supplementary file 9 — Figure S4 [file 41419_2025_8208_MOESM9_ESM.tif]

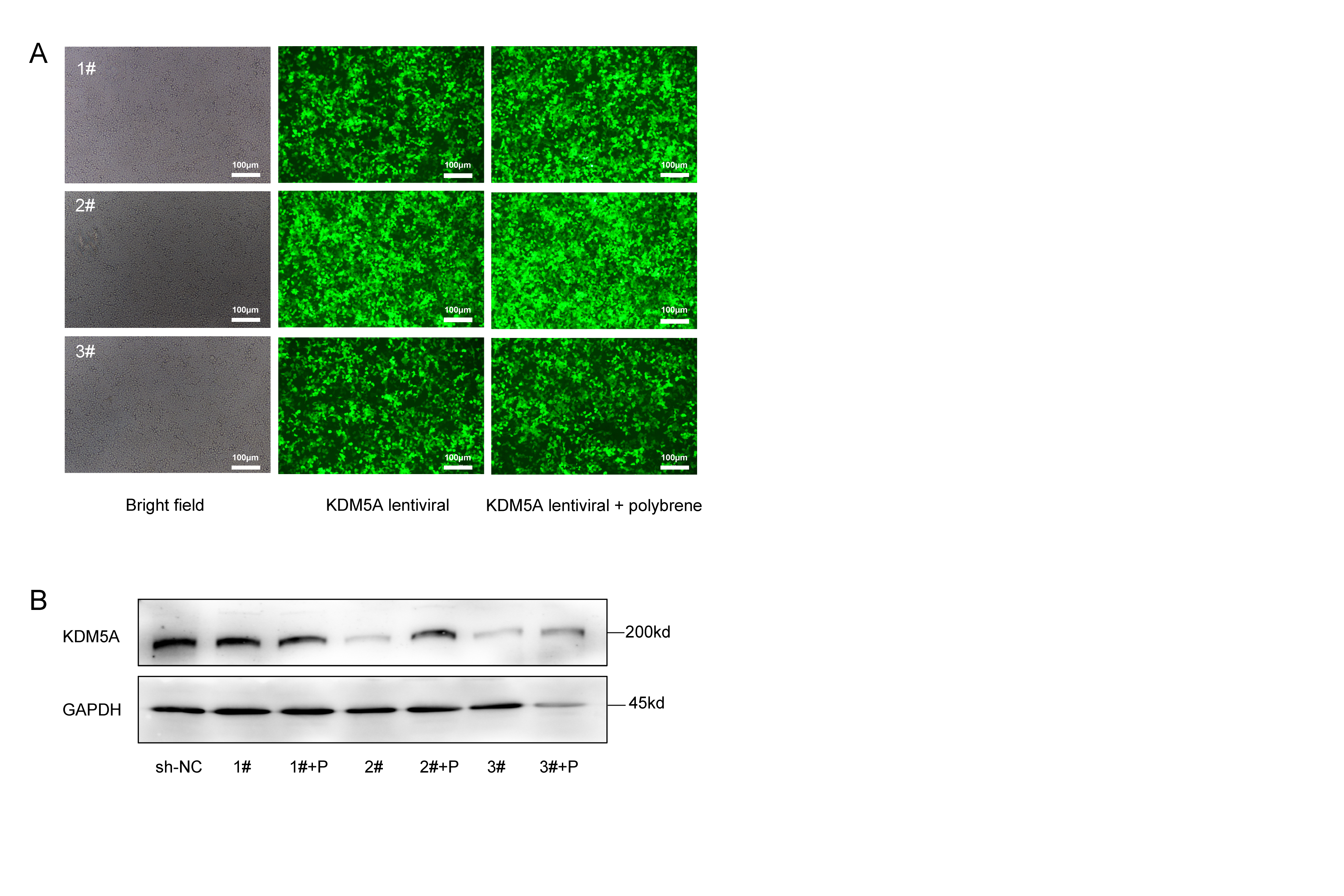

Supplement: Supplementary file 10 — Figure S5 [file 41419_2025_8208_MOESM10_ESM.tif]

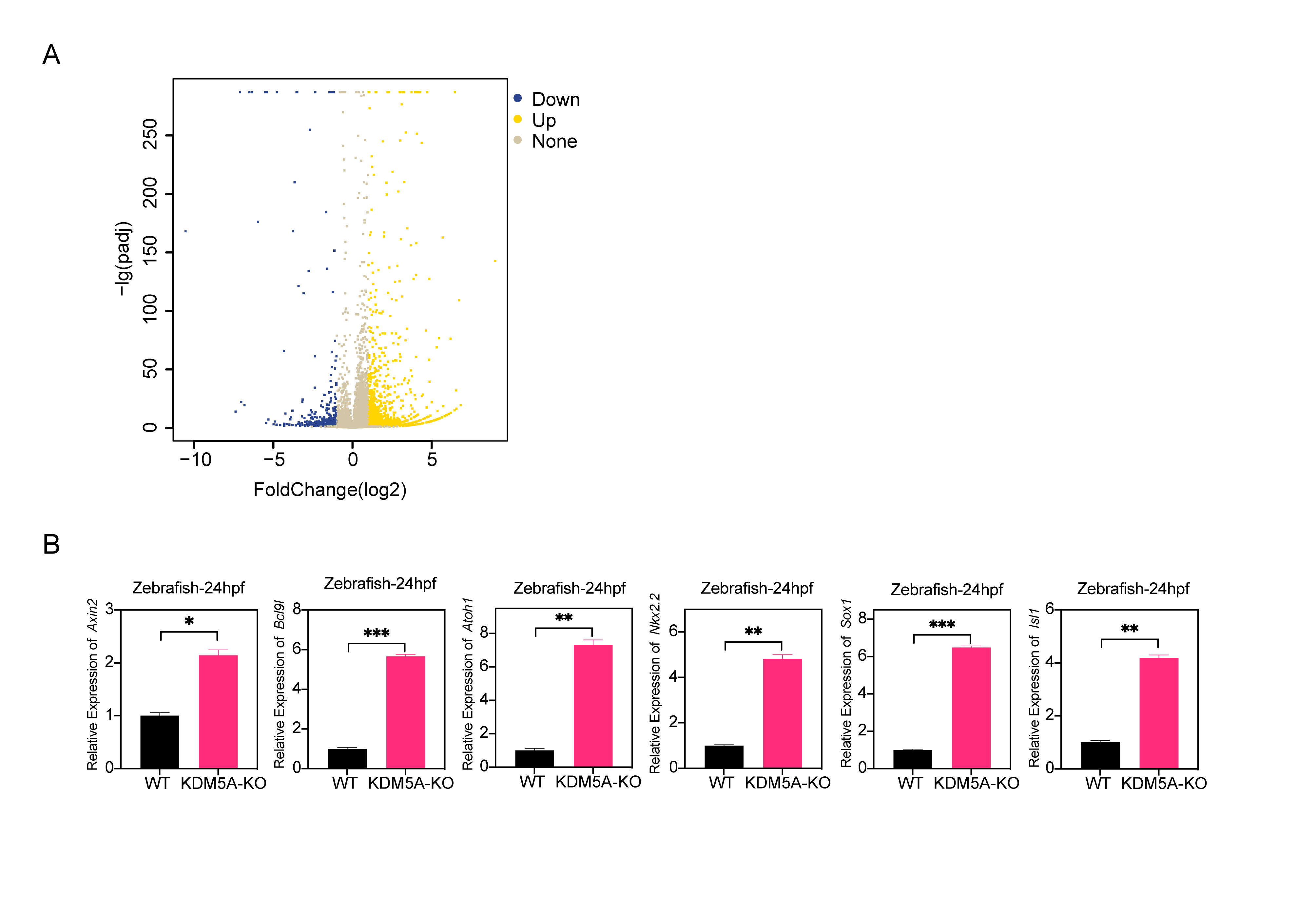

Supplement: Supplementary file 11 — Figure S6 [file 41419_2025_8208_MOESM11_ESM.tif]

Fig2B

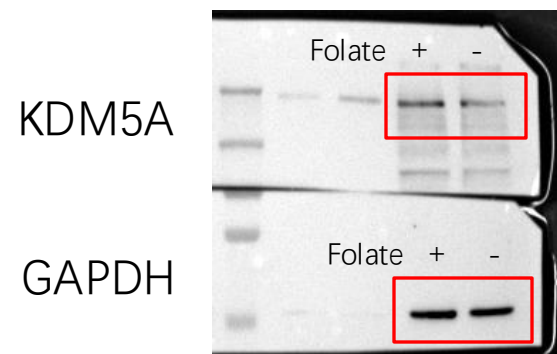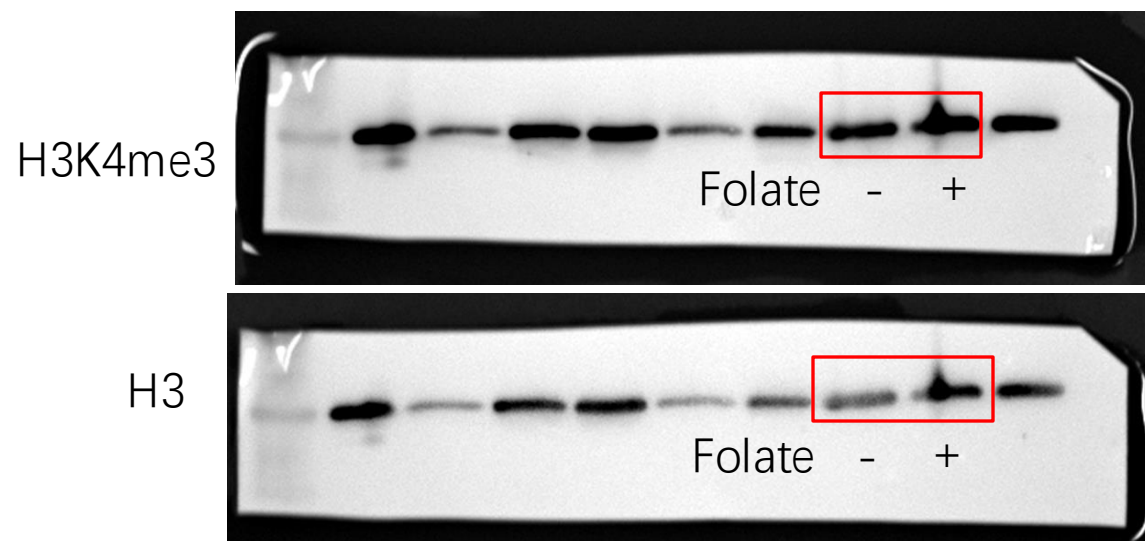

Fig3A

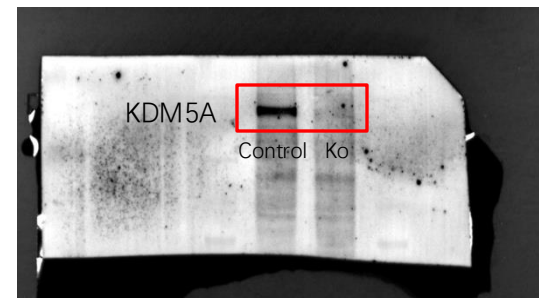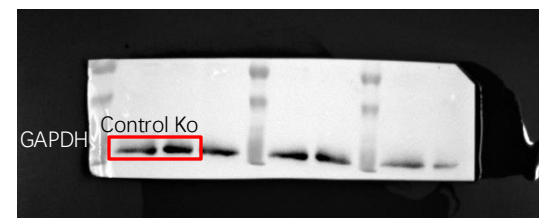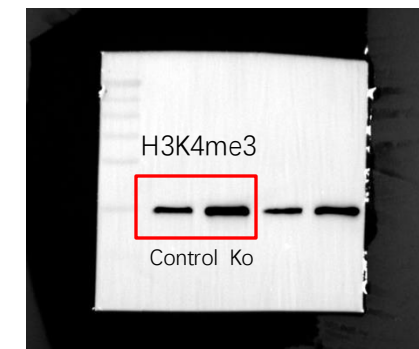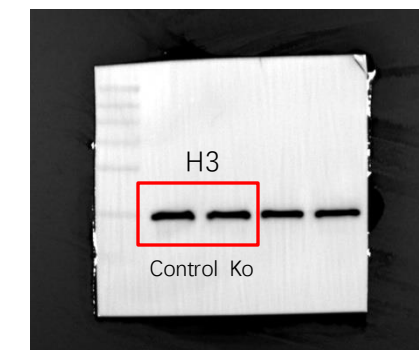

Fig4E

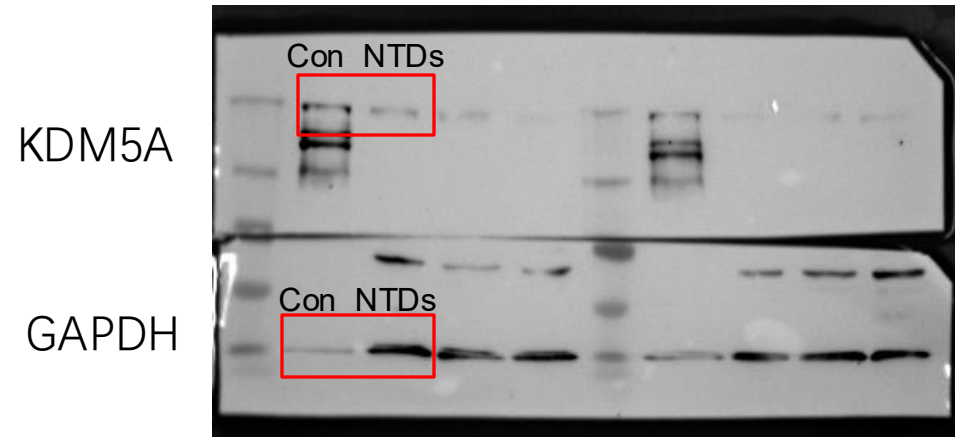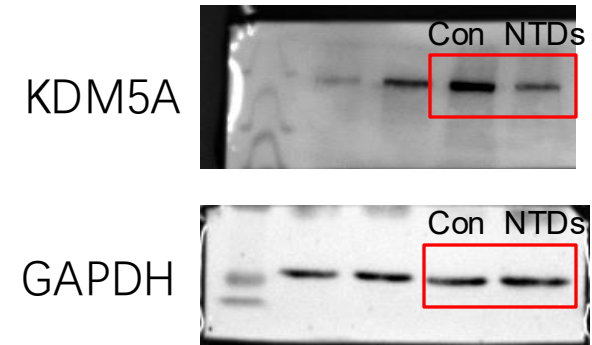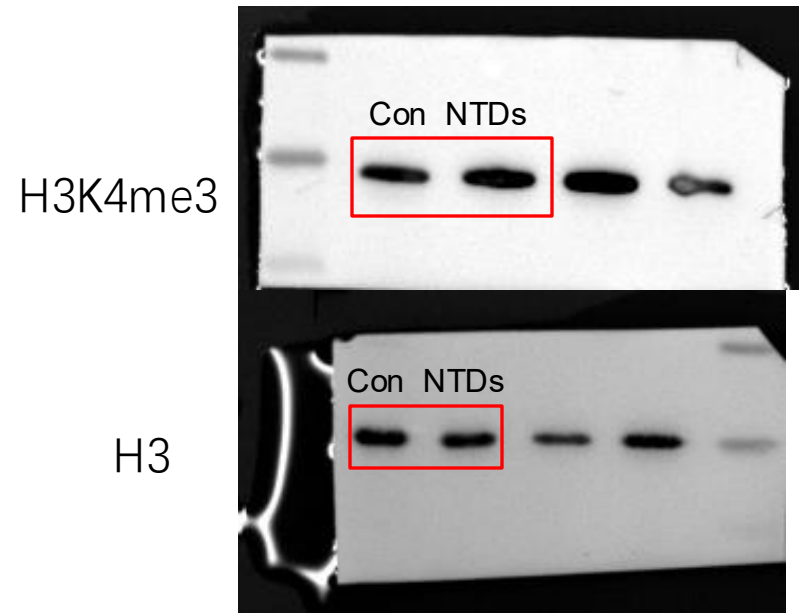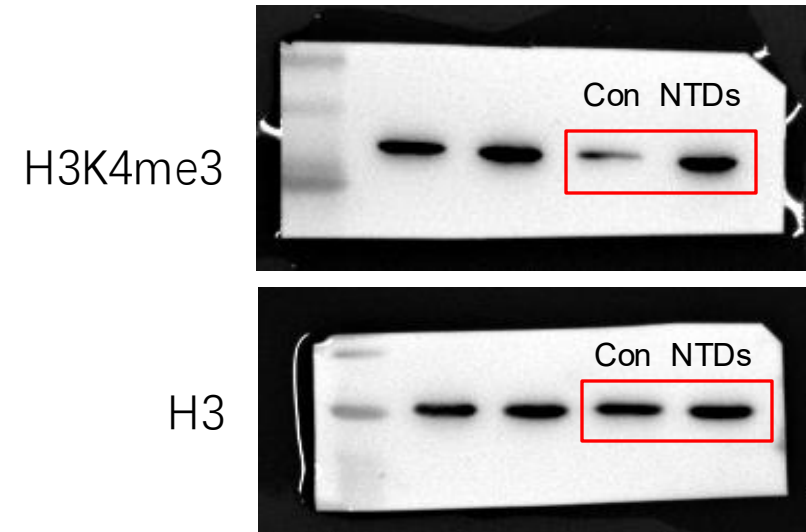

Fig5B

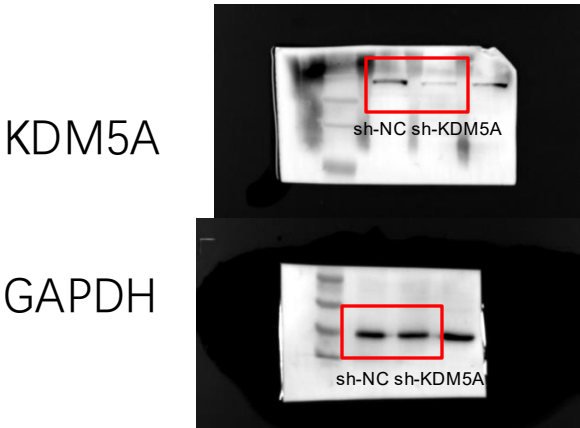

Fig7D

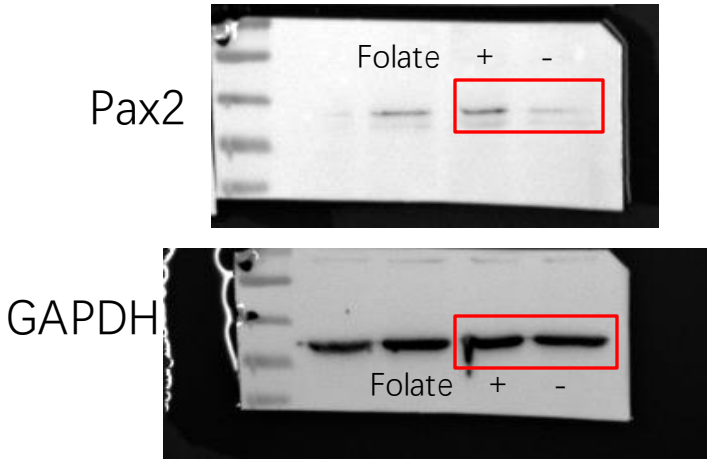

Fig7J

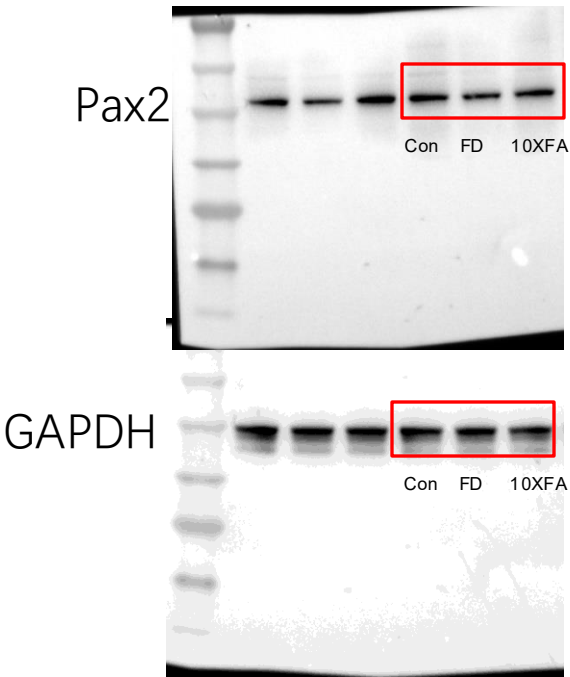

Fig8E

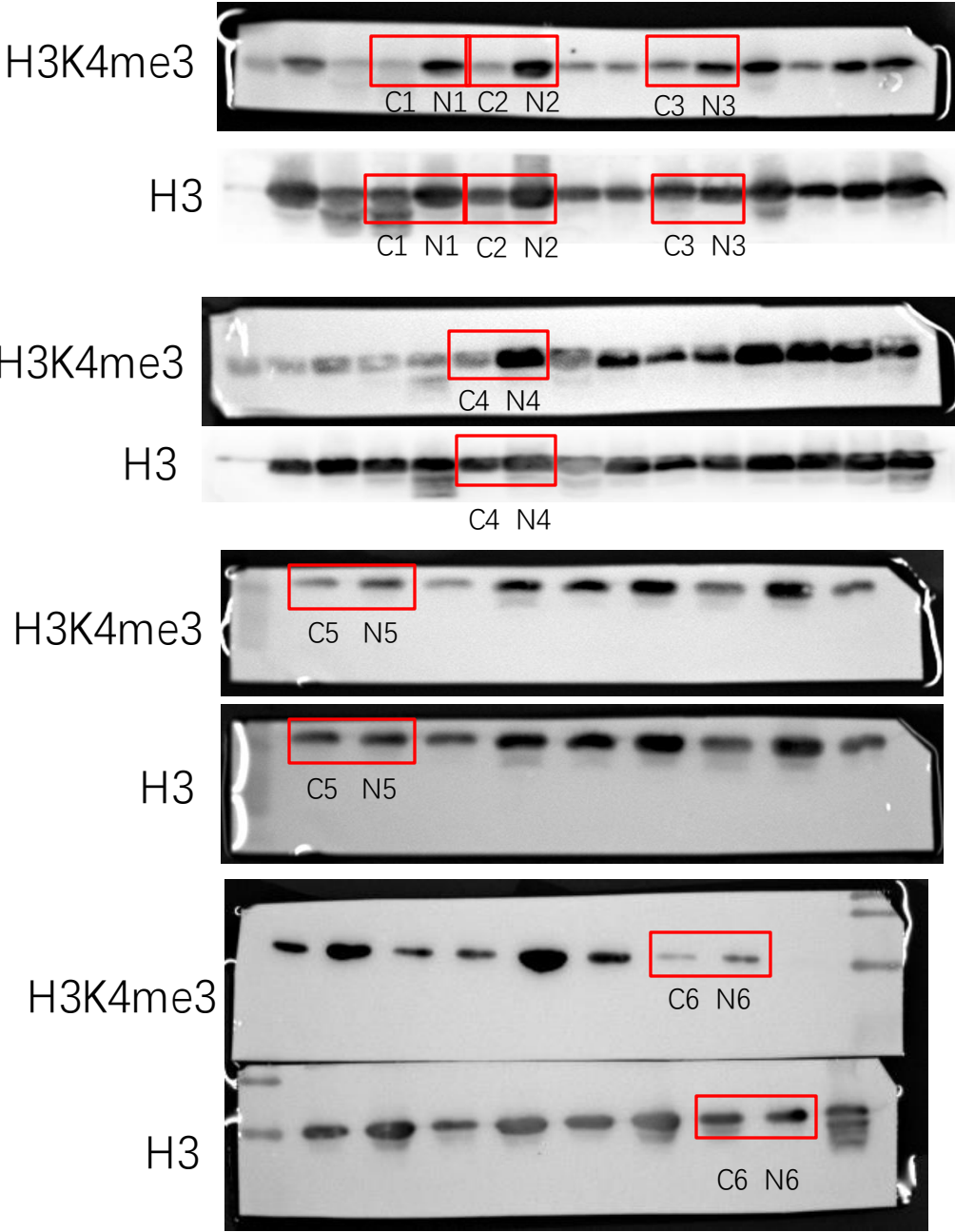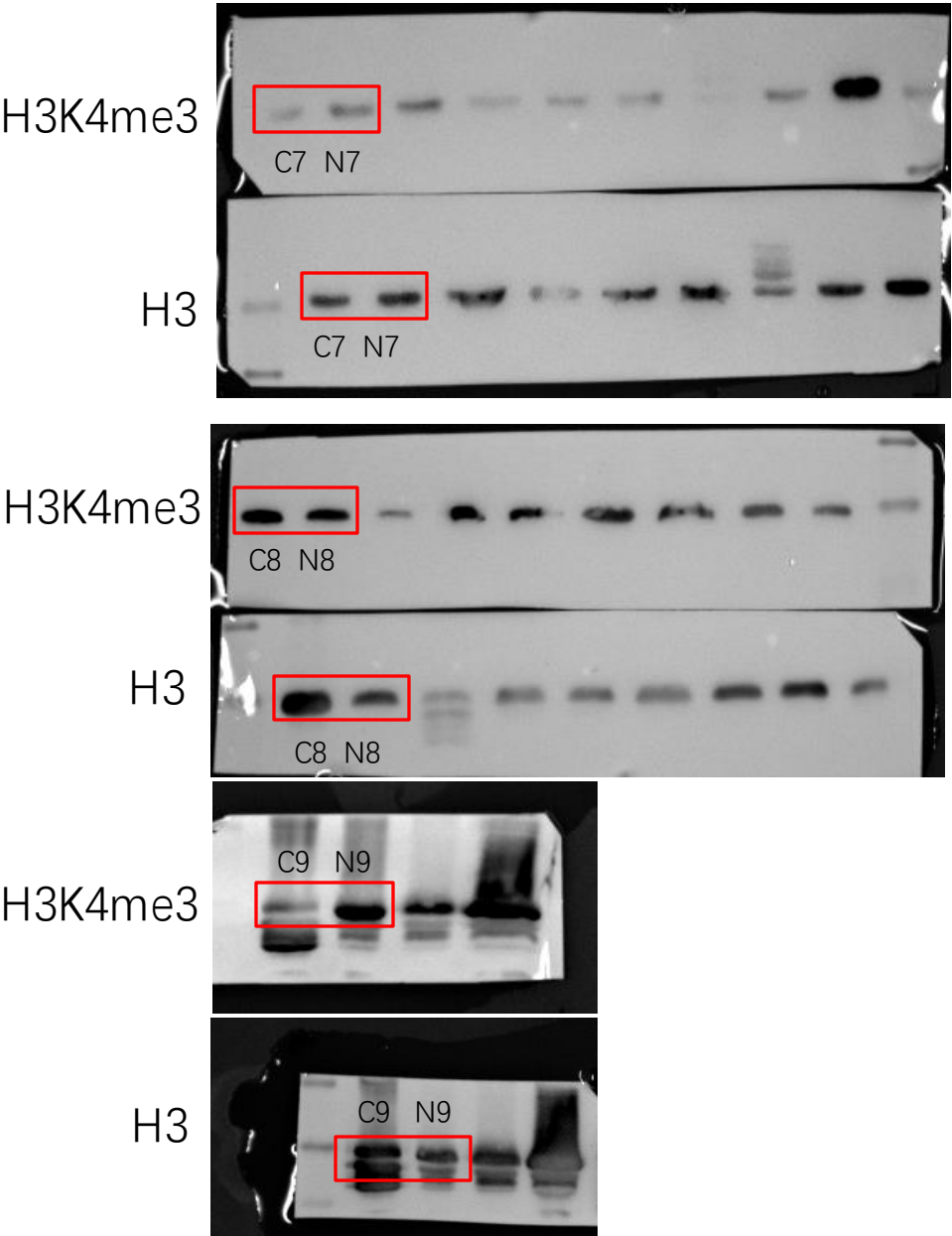

FigS2A

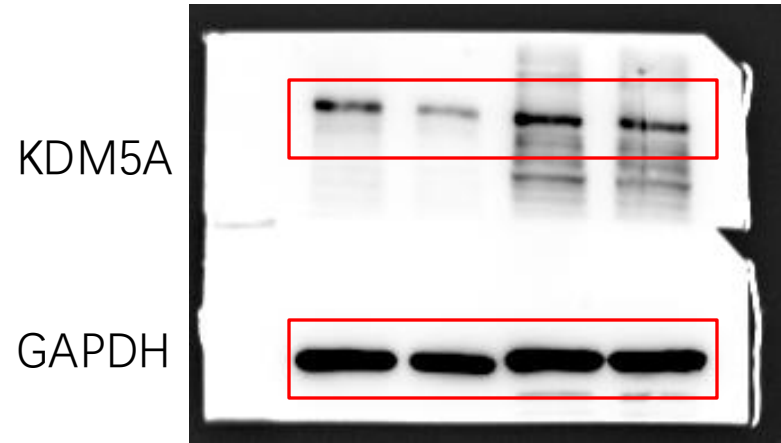

FigS2B

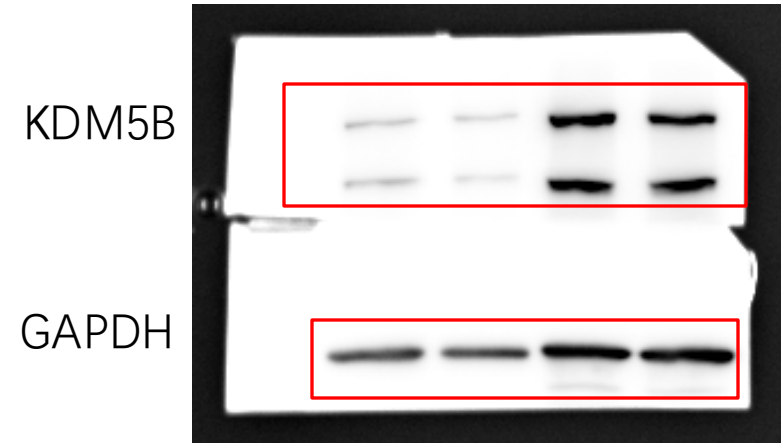

FigS2F

KDM5A

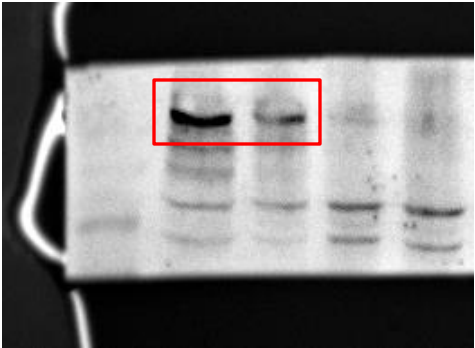

GAPDH

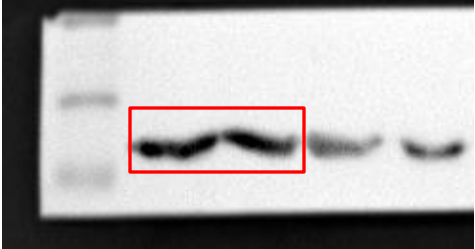

H3K4me3

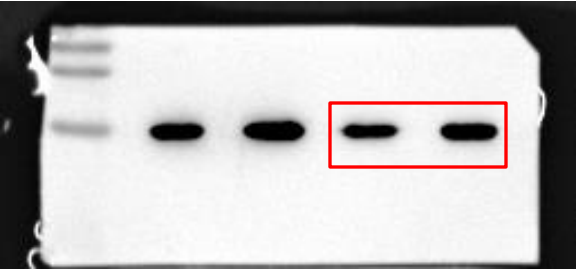

H3

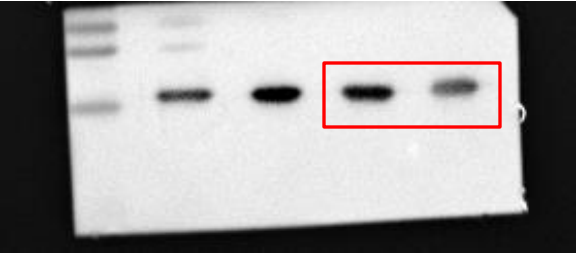

FigS2G

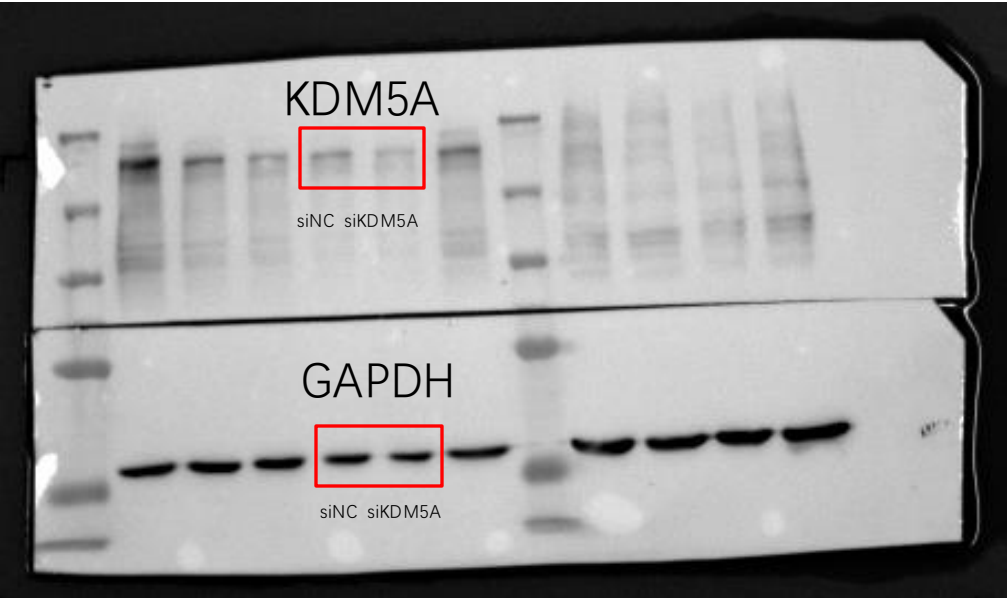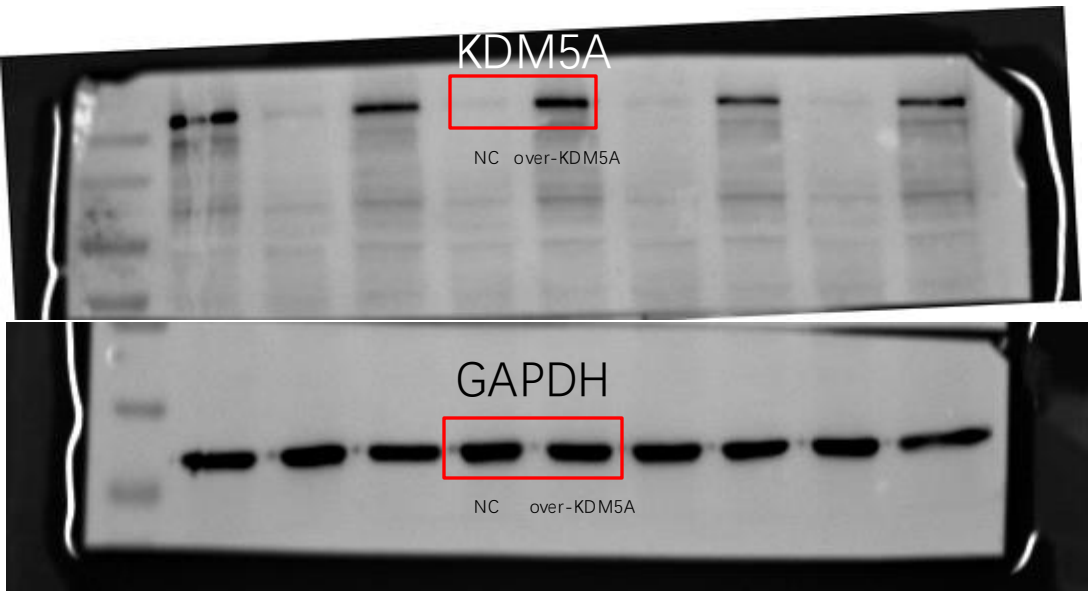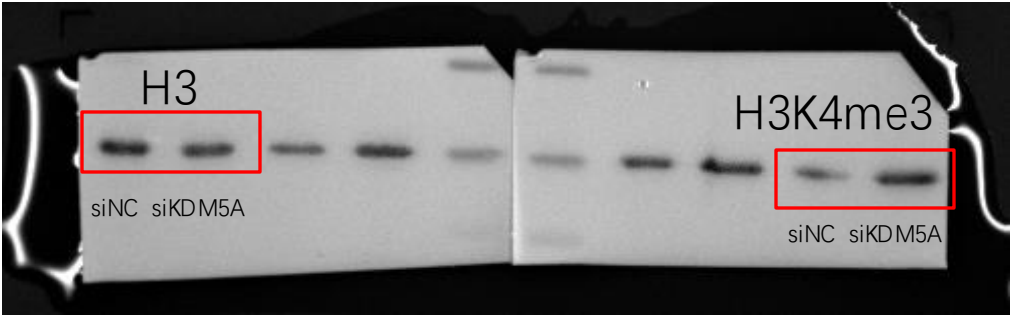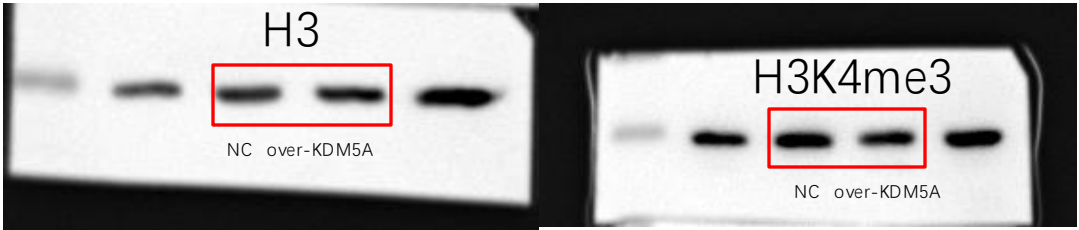

FigS5B

KDM5A

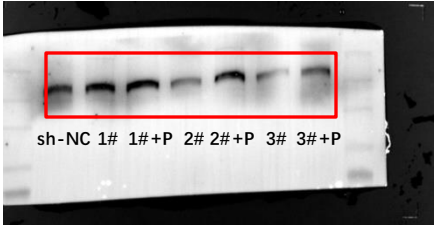

GAPDH

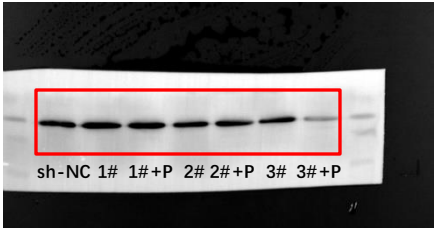

Supplement: Supplementary file 12 — uncropped WB image [file 41419_2025_8208_MOESM12_ESM.pdf]
